# Using the Multicomponent Aerosol FORmation Model (MAFOR) to Determine Improved VOC Emission Factors in Ship Plumes

**DOI:** 10.3390/toxics12060432

**Published:** 2024-06-14

**Authors:** Lea Fink, Matthias Karl, Volker Matthias, Andreas Weigelt, Matti Irjala, Pauli Simonen

**Affiliations:** 1 Helmholtz-Zentrum Hereon, Department of Coastal Environmental Chemistry, 21052 Geesthacht, Germany; lea.fink@hereon.de (L.F.); volker.matthias@hereon.de (V.M.); 2 Bundesamt für Seeschifffahrt und Hydrographie, 20359 Hamburg, Germany; andreas.weigelt@bsh.de; 3 Aeromon Oy, FI-00240 Helsinki, Finland; matti.irjala@aeromon.io; 4 Faculty of Engineering and Natural Sciences, Tampere University, FI-33720 Tampere, Finland; pauli.simonen@tuni.fi

**Keywords:** VOC emission factor, ship emissions, aerosol box model

## Abstract

International shipping’s particulate matter primary emissions have a share in global anthropogenic emissions of between 3% and 4%. Ship emissions of volatile organic compounds (VOCs) can play an important role in the formation of fine particulate matter. Using an aerosol box model for the near-plume scale, this study investigated how the changing VOC emission factor (EF) for ship engines impacts the formation of secondary PM_2.5_ in ship exhaust plumes that were detected during a measurement campaign. The agreement between measured and modeled particle number size distribution was improved by adjusting VOC emissions, in particular of intermediate-, low-, and extremely low-volatility compounds. The scaling of the VOC emission factor showed that the initial emission factor, based on literature data, had to be multiplied by 3.6 for all VOCs. Information obtained from the box model was integrated into a regional-scale chemistry transport model (CTM) to study the influence of changed VOC ship emissions over the Mediterranean Sea. The regional-scale CTM run with adjusted ship emissions indicated a change in PM_2.5_ of up to 5% at the main shipping routes and harbor cities in summer. Nevertheless, overall changes due to a change in the VOC EF were rather small, indicating that the size of grid cells in CTMs leads to a fast dilution.

## 1. Introduction

Marine transport emissions contribute considerably to global air pollution, e.g., [1,2,3]. Ships have the potential to decline the air quality in coastal regions because roughly 70% of ship emissions occur within a distance of 400 km from land [4]. According to Klimont et al. [5], the proportion of international shipping’s particulate matter primary emissions to global anthropogenic emissions is between 3% and 4%, which is comparable to road traffic. Through a combination of chemical reactions and microphysical processes involving precursor pollutants, secondary pollutants are formed in the atmosphere. Secondary atmospheric particulate matter is formed through chemical reactions in the atmosphere involving gaseous precursors.

There is evidence linking PM_2.5_ (particulate matter < 2.5 µm) exposure to the development of certain lung conditions, cancer, or type 2 diabetes [6,7,8]. These small particles can penetrate deeply into lung alveoli, by inhalation, and cause important adverse effects on human health [9,10,11]. The smaller the particle, the easier and deeper it can enter into pulmonary systems [12]. Long-term exposure to PM_2.5_ was identified to lead to decreased life expectancy, early death, and morbidity [13,14,15]. Further, there is no safe threshold for PM_2.5_ concentrations according to the WHO [16].

Volatile organic compounds (VOCs) play an important role as precursors for the formation of fine particulate matter in the atmosphere [17,18]. Most of the particle growth is caused by organic compounds in the gas phase [19,20]. VOCs react in the atmosphere with atmospheric oxidants such as the hydroxyl (OH) radical, the nitrate (NO_3_) radical, ozone (O_3_), and chlorine (Cl) atoms [17]. The initial oxidation step produces alkyl or substituted alkyl radicals by the H-atom abstraction from C–H bonds or the addition to C=C bonds. The alkyl radicals react with molecular oxygen (O_2_) to give organic peroxy radicals, which subsequently can react with nitrogen oxides (NO_x_), originating from various emission sources, to produce ozone under sunlight. In the fresh exhaust of diesel engines, most primary organic particulate emissions are semi-volatile [21]; thus, to a certain extent, they evaporate accompanied by atmospheric dilution, generating large amounts of low-volatility gas-phase material that is, after several oxidation steps, available for later condensation to particles [22]. Photo-oxidation initiates the formation of particles, as it results in the generation of abundant precursors for the nucleation and growth of fine particles in air [23,24]. Experiments in the laboratory indicate that photo-oxidation of diesel exhaust rapidly produces organic aerosols [22]. Oxidation of gas-phase organic species and chemical aging leads to the production of secondary organic aerosol (SOA) [25,26,27,28]. The SOA is, in large part, responsible for the formation of fine particulate matter [25,29]. SOA contributes 20% to 60% globally and, during periods with high pollution, up to 30% to 77% to total PM_2.5_ [30,31,32,33]. Particularly in summer, the aerosol mixing ratios in the Mediterranean area are higher than those in most continental European regions [34].

Previous modeling studies have shown the relative potential ship impact on PM_2.5_. In Karl et al. [35], a regional study on the impacts of ship emissions in the Baltic Sea region was done using three regional-scale chemical transport models. According to that study, the average contribution of ships to PM_2.5_ levels varied from 4.15% to 6.5% over the whole Baltic Sea region and from 3.15% to 5.7% along the shore. Studies regarding the Mediterranean region found a relative ship impact on the PM_2.5_ concentrations of a share of 5% to 20% of the total PM_2.5_ concentrations, e.g., [36,37,38].

Regional-scale chemistry transport model (CTM) systems tend to underestimate the measured PM (particulate matter) [38,39]. This can be traced back to the relatively coarse grid resolution in regional-scale models that do not account for chemical transformation mechanisms in sub-grid pollution plumes. Although regional CTM systems generally contain chemically speciated PM_2.5_, they do not divide the PM into smaller particles nor consider ultrafine particles (UFPs) with aerodynamic diameters of <0.1 µm [40]. While UFPs have a small contribution to particle mass, the physical processes transforming them are highly dynamic and of great importance to a detailed investigation of PM formation. The physical transformation of ultrafine particles rapidly changes the ambient PNC (particle number concentration) due to nucleation, condensation, evaporation, coagulation, and dry deposition [40]. Through particle growth, a sub-set of these particles (larger than about 50–80 nm) can produce cloud condensation nuclei (CCN), influencing cloud formation and the radiative balance [41].

Gonzales et al. [42] found inland transport of UFPs from ship plumes in Santa Cruz de Tenerife, with ship emissions having a share of 65% to 70% of the UFP concentrations. Pirjola et al. [43] also reported an increase in UFP concentrations due to ship emissions in European coastal cities. Secondary volatile particles make up a large fraction of the UFPs that form in the nucleation of gas-phase species and grow fast in size by condensation of organic vapors and sulfuric acid. Shipping is a likely source of secondary particle formation because of the high sulfur content of the fuel oil they burn [44]. Particles coming from ship exhaust contain several chemical compounds such as particulate sulfate, black carbon, organics, ash, and heavy metals, each having an impact on the properties of particle emission [42,45]. Due to this, particle size distribution in ship plumes can range from 5 nm to >3 µm [3]. Still, few studies have been conducted to investigate the change in particle characteristics in the exhaust plume of ships directly in the stack and in parallel in aged plumes on land [46]. Karl et al. [47] estimated the exposure to UFPs from ship exhaust in major harbor areas in two cities in Finland by measuring and modeling the dispersion and dynamics of aerosols. They found increased UFP concentrations due to ship emissions up to a distance of 3600 m. According to a study conducted in the port area of Marseille, the mean potential impact from ships in July 2020 was 6% to 9% for SOAs and 27% to 51% for total particle number concentrations [48].

Ship plume parametrizations on plume dispersion and chemistry in plumes have been evaluated in previous studies, e.g., [49,50,51], but usually not for aerosols.

To describe the development of the particle number and mass size distribution from the point of emission to the site of interest, the modeling of particle transformations in parallel with plume dispersion is required. It is crucial to evaluate the evolution close to the source with high spatial and temporal resolution since the particle size and composition changes fast after exhaust release [52]. Lagrangian size-resolved aerosol models can be used for investigations when higher resolutions in time and space are necessary.

VOCs are considered to be important for the exhaust plume development and the size distribution, since they might condense shortly after they enter the atmosphere from the emission source, depending on their volatility. Despite the fast condensation during the dilution of the hot exhaust plume, they can still reach gas-phase saturation in the expanding plume, due to the high amounts of VOCs present in ship exhaust. Only a few studies focus on VOCs in ship exhaust. The shift from high-sulfur residual fuel oil to low-sulfur diesel or heavy oils that are often richer in short-chain hydrocarbons was shown to have a major impact on the rise in VOC emissions [53]. The VOC mixture emitted from ships tends to produce more secondary organic aerosol than diesel and gasoline vehicles, while the ozone-forming potential is comparable. The different contents of organic components with high molecular weight is the major reason for this. Ship exhaust contains more heavy VOCs than automotive exhaust (i.e., alkanes and aromatics). Aromatic hydrocarbons play a significant role in SOA formation [54].

The goal of the present study was to simulate particle transformation within aging ship plumes that were observed in a measurement campaign within the SCIPPER project. The aim of this project was to obtain further insights in the capability of several monitoring approaches for shipping emissions (https://www.scipper-project.eu/, accessed on 12 May 2024). The in-plume particle evolution was simulated with an aerosol box model, ship emissions were adjusted, and simulations with the adjusted emissions were performed using a larger-scale CTM. VOC emission factors (EFs) for ships were used to establish a link between the box model and the regional-scale model. Two related questions to be answered in this study are (1) how the changing VOC emission factors for ships impact the formation of particulate matter and (2) how the output of a box model for the near-plume scale can be integrated into the regional-scale CTM. The sectional aerosol box model MAFOR v2.1.0 (Multicomponent Aerosol FORmation model) was applied in the present study [52,55] to simulate the particle size distribution between the ship stack and the surroundings for up to 1 h travel time downwind [47]. Furthermore, in the applied model, gas-phase chemistry is coupled with aerosol dynamics. For the model input, measurement data of a ship exhaust plume measured on board a passenger ferry operated in the Baltic Sea, and air pollutant concentrations and meteorological data, both measured 500 m distant from the shipping lane, were used. The simulations of the field campaign data were used to adjust the VOC emission factors in the ship emission dataset STEAM (version 3.3.0.) of the Finnish Meteorological Institute (FMI) [56,57,58,59]. STEAM ship emissions were further used as input for regional-scale model simulations with the Community Multiscale Air Quality Model (CMAQ) [60]. CMAQ was applied as described in Fink et al. [38,61] on a model domain covering most of the Mediterranean Sea. The Mediterranean Sea was in the focus due to the higher insolation and SOA formation potential compared to the Baltic Sea, leading to a stronger response and better possibility for validation.

## 2. Materials and Methods

### 2.1. Measurements on Board and on Land

The MAFOR model runs were initialized by using measurement data as input data. The measurements of ship emissions were done on board a passenger ferry, which is a Passenger/Ro-Ro Cargo Ship powered by two four-stroke Wärtsilä 8ZAL40S engines with a maximum continuous rating (MCR) of 6000 kW and a nominal speed of 510 rev/min. All primary engines have SCR catalysts and are converted to dual-fuel engines that can run either on methanol or regular marine fuels. The used fuel from plumes investigated in the current study was marine gas oil (MGO). Carbon dioxide (CO_2_), nitrogen monoxide (NO), nitrogen dioxide (NO_2_), sulfur dioxide (SO_2_), carbon monoxide (CO), and ammonia (NH_3_) as initial gases in the ship plume were measured in the ship stack by an Aeromon BH-12 measurement device [62]. The uncertainty for NO_x_ measured by the Aeromon BH-12 is in a range of ±19.7%. The measurement device was located in the stack after the catalyst [63].

Particle number and mass size distributions from the passenger ferry were measured at a resolution of one second by an Engine Exhaust Particle Sizer (EEPS; size distribution: 5.6 nm–560 nm) and an Electrical Low Pressure Impactor (ELPI; size distribution of 7 nm–9.9 µm). The measurement point for these instruments was in the stack after the Selective Catalytic Reduction (SCR) and it was measured downstream of a combination of a porous tube diluter and ejector diluter [63].

#### Ship Plume Event

**Figure 1:**
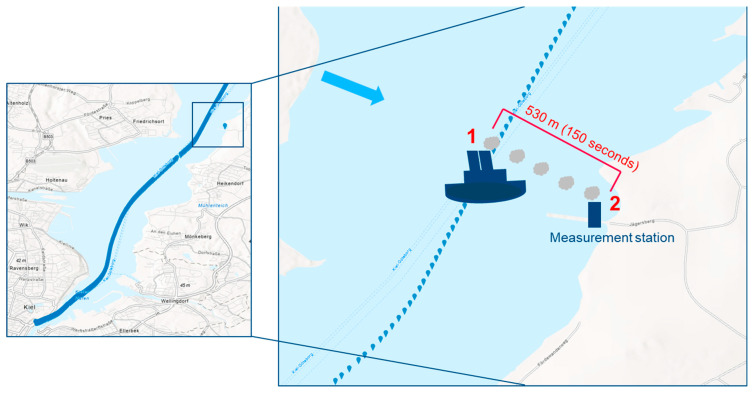
Shipping lane (blue dots), measurement station = mobile lab (single blue square) and the wind direction (284° ± 15°, blue arrow). The distance from the ship lane to the mobile laboratory is 530 m.

**Figure A1:**
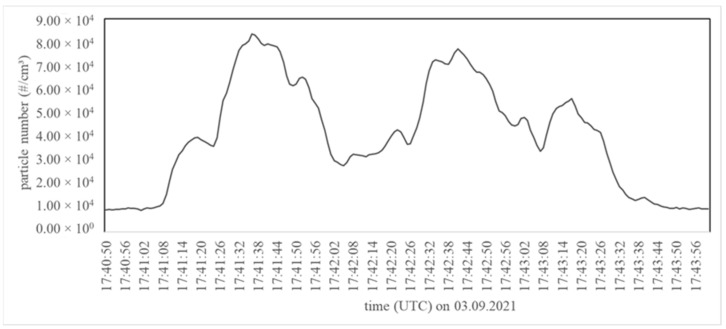
Total particle count measured during the SCIPPER measurement campaign on 03.09.2021 in Laboe at the Kieler Förde (54.392809, 10.209186). The plume arrived at the measurement station at 17:41:37 UTC and the plume peak was determined when the total concentration was highest. The uncertainty for the total particle number is approximately ±10 %, as reported for the German Ultrafine Aerosol Network [103].

The ship plumes were from a regular passenger ferry that travels between Kiel and Gothenburg. All captured plumes originated from that vessel. The ship plume used as a reference for the aerosol formation model MAFOR was detected during the SCIPPER measurement campaign (from 31 August 2021 to 6 September 2021) on 3 September 2021 in Laboe at the Kiel Fjord (“Kieler Förde”) (54.392809, 10.209186; Figure 1). During that event, both onboard and on-shore observations were available. Thus, this plume is used as a reference and all adjustments in the configuration of MAFOR simulations were done based on this occurrence. The plume arrived at the mobile laboratory at 17:41:37 UTC. The evolution of the plume and the decrease in particle number over time was used to determine the time of the plume peak (i.e., when the total concentration at Laboe was highest; Figure A1) and the time when only background particles were measured. The wind direction was 284° (±15° in one hour). If not indicated otherwise, the term ship plume hereafter refers to the reference plume.

For comparison with this reference plume, an additional 12 on-shore plume events from the same ferry between 2021 and 2023 detected at the mobile lab were evaluated in the present study. The plume events were chosen when (i) measured in the afternoon between 16:00 UTC and 18:00 UTC, (ii) departing from Kiel, and (iii) measured in the time frame of mid-July to mid-October. A detailed overview is given in Table A1. The ship emissions were assumed to be the same as in the base case because the ferry passes the measurement location always at the same speed and engine load.

On-shore measurements of particles, gases, and meteorological parameters were carried out at the mobile lab at Laboe close to Kiel. For measuring the particles on shore, a Fast Mobility Particle Sizer (FMPS spectrometer Model 3091; size distribution: 5.6 nm–560 nm, manufacturer: TSI Incorporated) was used for small particles. Larger particles were measured by an OPS (Optical Particle Sizer Model 3330; size distribution: 300 nm–10 µm, manufacturer: TSI Incorporated). The time resolutions for FMPS and OPS were one second. The used air intake system for both particle measurement devices was the Sampling System for Atmospheric Particles from TSI for particles up to 10 µm. The plumes were detected automatically.

**Figure A2:**
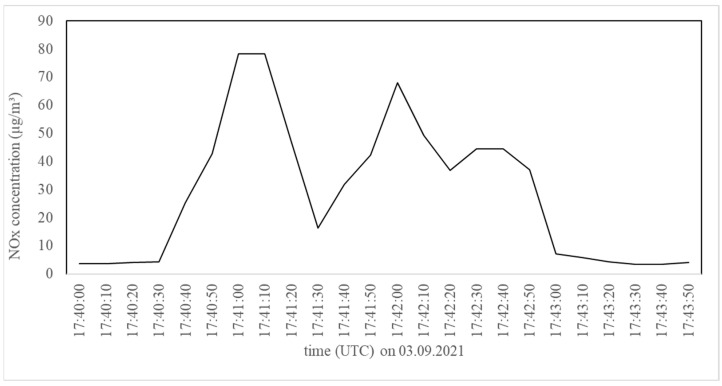
NO_x_ concentrations after the Germanica passed the measurement station. Gases and meteorological parameters were measured by an airpointer 4D measurement system for NO_x_, O_3_, SO_2_, and a weather station during the SCIPPER measurement campaign on 03.09.2021 in Laboe at the Kieler Förde (54.392809, 10.209186). The uncertainty for NO_x_ measurements is given by the manufacturer and is 2 µg/m^3^ (1 ppb) for measured concentrations <500 ppb.

Gases and meteorological parameters were measured with an airpointer 4D measurement system (manufacturer: JCT NextGen AQMS mlu-recordum) for NO_x_, O_3_, SO_2_, and a weather station. The lower detection limits are 0.4 ppb for NO_x_ and 0.5 ppb for O_3_ and SO_2_. The uncertainty for NO_x_ measurements is given by the manufacturer and is 1 ppb (2 µg/m^3^) for measured concentrations <500 ppb. The peaks of measured NO_x_ concentrations after the passing of the ships are shown in Figure A2.

### 2.2. MAFOR

#### 2.2.1. Input Data and Model Configuration

**Figure 2:**
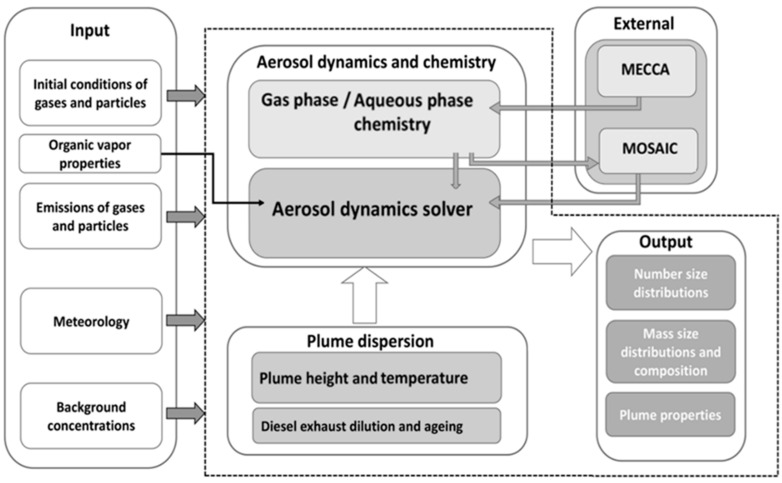
Illustration of the model structure, as presented in Karl et al. [52]. Dashed outline contains the MAFOR model. External modules are MECCA v4.0 and MOSAIC solver (the models are not part of MAFOR but included in the model distribution).

The open-source model MAFOR v2.1.0 was used in the present study. It is a zero-dimensional Lagrangian-type sectional aerosol box model [52,55] available on Github: https://github.com/mafor2/mafor, accessed on 15 May 2024. The model calculates the development of particles in terms of particle number and mass as well as changes in particle average density. The model was run in plume dispersion mode to display a single exhaust plume along one dimension in space. All simulated particles are assumed to be spherical. The model is linked to the MECCA [64,65] chemistry module, which contains up-to-date photolysis rates for VOC chemistry (Figure 2).

The model relies on several input data files that include general data (meteorological data, time, and location), configuration (processes that are used in the model), initial aerosol size distribution (in terms of mass concentration), gas-phase emission (concentration) and background aerosols (particle mass concentration), organics (properties of the organic vapors), and dispersion (plume dispersion and deposition parameterization). The gas measurements and meteorology data obtained at the mobile laboratory on shore were used in the model and defined the air parcels’ trajectory. 

The following processes were considered in the MAFOR run: dry deposition over the water surface [66], nucleation, condensation of organics and of water, and coagulation. The coagulation of particles by Brownian diffusion was considered. The dilution of the plume through expansion into the background air with a low particle concentration was done with the dispersion parameterization for ship plumes under convective conditions in the open sea.

The plume dilution after MAFOR model initiation followed the parametrization of Chosson et al. [67] for the convective boundary layer to match the modeled concentrations in the ship plume. The parameters for plume dispersion were not adjusted and used as provided in the MAFOR model. The parameters for plume dispersion in the MAFOR model and the original parameters of Chosson et al. [67] are listed in Table A2. The plume height approximation follows a formulation of von Glasow et al. [68] (Table A3).

The nucleation mechanism of homogeneous H_2_SO_4_–H_2_O [69,70] was applied in the MAFOR simulation. For this, the consideration of H_2_SO_4_ is of importance [71]. Starting concentrations for H_2_SO_4_ in the stack were set according the concentrations received from Karl et al. [47], with a value of 0.1 × 10^11^ cm^−3^.

The EF of NO_x_ was measured on board by the Aeromon BH-12 measurement device [61] in the exhaust stack and was used to calculate the EF for other gases. The initial guess for NO_x_ EF was based on Fridell et al. [72].

The measurements were too close to the emission source for the MAFOR model to start (it starts usually 1 s after the plume left the stack); therefore, a dilution ratio for the gases of 1:8 was used as described in Karl et al. [47]. Karl et al. [47] determined that the plume reached the background temperature after 1 s, when the nucleation was complete and was, therefore, no longer taken into account. Therefore, they only considered the error due to neglecting coagulation during exhaust cooling. In the present study, the simulation of the plume also started after the first second from the point of release; therefore, the initial condensation effect during cooling was not considered. The particle instruments measuring the size distribution in the hot exhaust did not detect significant numbers of particles <7 nm diameter, probably due to the lower size cut-off at 5.6 nm. The SOA concentrations were simulated using a hybrid method of condensation/evaporation and absorptive partitioning into an organic liquid (according to Kerminen et al. [73]) and using a 2D VBS set [74] consisting of nine organic compounds with different volatility. The simulations included the basic gas-phase reactions in the troposphere and the Mainz Organic Mechanism (MOM) as an oxidation scheme for VOCs. The chemistry of the following VOCs were considered in the simulations: ethane (C_2_H_6_), ethylene (C_2_H_4_), propane (C_3_H_8_), trimethylbenzene (TMB), toluene (TOL), xylene (XYL), and primary emitted organic vapors, such as long-chained n-alkanes, which are represented by PIOV (intermediate volatility), PSOV (semi-volatile), and PELV (extremely low-volatile). Reactions were taken from MECCA [64,65].

To start the MAFOR simulation, the particle mass initial at the stack was calculated from the measured particle number. For this, the particles in sizes from 6.04 nm to 523.3 nm measured by EEPS (32 bins) and larger particles with sizes from 764.4 nm to 6.3 µm (4 bins) measured by ELPI were used. The resulting 36 bins were assigned to the four modes in MAFOR (NU: nucleation mode, AI: Aitken mode, AS1: accumulation mode 1, AS2: accumulation mode 2), as described in the following, and the mass was calculated as a sum within those by assuming a density of 1.0 g/cm^3^. The fourth mode of the measured size distribution at the ship stack is a second accumulation mode rather than a coarse mode; therefore, it was divided into a first and second accumulation mode. The median of in total 366 measurements of particle number and mass on board the ship on 03.09.2021 between 17:49:00 UTC and 22:54:30 UTC was calculated for each bin to determine the input data for the ship plume aerosol. This time window for measurements on the ferry was chosen according to the engine load and fuel used. At this time, the exhaust plume characteristics were expected to be the same as in the plume that came out of the stack at the time when the ship passed the measuring station. Measurements at the exact time of the passing were not available, but they were a few minutes thereafter. Therefore, the median from onboard measurements was used. The received measured aerosol mass was transferred into a MAFOR input file that provides the initial aerosol size distribution of the ship plume. The total aerosol mass concentration was divided into the four modes (NU, AI, AS1, AS2). The aerosol initial mass composition was subsequently converted to particle number based on the material densities of the different aerosol components, assuming spherical particles to ensure consistency in terms of mass and number [52]. The calculation of particle number and mass concentrations in the model was then done using a sectional size representation of the aerosol. The initial chemical composition in the ship plume was based on data from the literature [47,53,72] (Table A4).

Within MAFOR, the total mass was divided into H_2_SO_4_, OC, NH_4_, NO_3_, MSAp (methane sulfonate), salt, POA (primary organic material), EC, and ash for the ship aerosol and the background aerosol (Table 1). The background composition was taken from Karl et al. [46]. The emissions measured on the ship and their chemical compositions for the reference plume were also used for the simulation of the 12 other ship plumes.

For background aerosols, the particle mass was calculated from the particle number measured at the on-shore mobile lab (from FMPS and OPS devices). To avoid any influence of ship emissions, the median of the measured particle number from half an hour before the arrival of the plume was calculated for the background aerosol data. The distribution of the plume aerosol and background aerosol mass concentrations in the four modes is displayed in Table 1. The mass was distributed to fit the particle number distribution of the respective measurement.

Because of multicomponent condensation and particle coagulation, the composition of particles in the size bins may vary over time. MAFOR calculates rapid changes in the aerosol size distribution every 0.1 s. The simulation of the ship plume starts one second after release from the ship’s stack. The distance from the stack to the mobile laboratory was 530 m (corresponding to 150 s travel time of the ship plume).

#### 2.2.2. Comparison Simulated against Measured Data

The comparison between modeled and measured data was done by comparing particle number concentrations and mean diameter for different size classes on which the measured and the modeled bins were mapped. These classes were set following the size class distribution in Karl et al. [52] by adapting the modeled size distribution with the size classes (bins) of the measured size distribution. The resulting size classes have the diameter ranges S1: 1–10 nm; S2: 10–20 nm; S3: 20–50 nm; S4: 50–100 nm; S5: 100–300 nm; S6: 300–600 nm; S7: >600 nm. The mean diameter of each size class was calculated as follows:

(1) $$\bar{d_{n}}=\frac{\sum_{i}{(N}_{i}\cdot d_{i})}{\sum_{i}N_{i}}$$

where *d_n_* denotes the mean diameter per size class, N_i_ is the number of particles, and d_i_ is the midpoint of the size class; i is running over the size bins, n is running over the size classes.

### 2.3. CMAQ and Ship Emission Dataset

The CMAQ model v5.2 was applied with configurations as described in Fink et al. [38,61] for four months (March, June, September, December) of 2015 on a model domain covering most of the Mediterranean Sea with a resolution of 12 × 12 km^2^. CMAQ calculates atmospheric concentrations as well as deposition fluxes of gases and aerosols based on emission input data [75,76]. The Carbon Bond 5 (CB05) chemical mechanism with updated toluene chemistry cb05tucl [77] including the chlorine chemistry extension (CB05-TUCL; https://www.airqualitymodeling.org/index.php/CMAQv5.0_Chemistry_Notes, accessed 21 June 2023) was used for the gas-phase chemistry. Secondary inorganic aerosols were formed using the AERO6 aerosol mechanism. The ISORROPIA model was applied for solving the gas-phase–aerosol partition equilibrium of sulfuric acid (H_2_SO_4_), nitric acid (HNO_3_), hydrochloric acid (HCl), and ammonia (NH_3_) [78,79]. Secondary organic aerosol (SOA) is formed from isoprene, terpenes, benzene, toluene, xylene, and alkanes [80,81]. CMAQ allows for dynamic mass transfer of semi-volatile inorganic gases to coarse mode particles, which facilitates the replacement of chloride by NO_3_^−^ in sea salt aerosols [82].

Kelly et al. [83] described the method for calculating sea salt emissions as used in this model configuration. Biogenic emissions (NMVOC from plant and soil NO) were previously calculated using the MEGAN Model v3 [84] and then combined with the land-based emissions. Windblown dust emissions and NO_x_ lightning treatment were not taken into account. For land-based anthropogenic emissions, the gridded emissions obtained from the CAMS-REG v2.2 emission inventory were used as input. Gridded emission files contain GNFR (Gridded Nomenclature for Reporting) emission sectors for each country for the air pollutants NO_x_, SO_2_, NMVOC, NH_3_, CO, PM_10_, PM_2.5_, and CH_4_. The detailed distribution and splitting of land-based emissions used for the CMAQ run is described in Fink et al. [38,61].

The ship emission dataset received from STEAM (version 3.3.0) [56,57,58,59] was used as input data for CMAQ. Mineral ash, CO, CO_2_, elemental carbon (EC), NO_x_, organic carbon (OC; assumed to be non-volatile), PM_2.5_, particle number count (PNC), sulfate (SO_4_), SO_x_ (containing SO_2_ and SO_3_), and VOC emissions were provided in two vertical layers (0 m to 36 m; 36 m to 1000 m). VOC emissions were split by FMI into four groups based on their characteristics as a function of engine load to limit the number of produced emissions and the computing resources required to run the STEAM model. The VOC groups contain reactive (volatile) VOCs as well as organic compounds of different volatility. VOC EFs in STEAM were calculated using average values from previous publications [85,86,87,88].

The COSMO model, version COSMO5-CLM16, was used to simulate the meteorological data for CMAQ [89,90]. The MCIP (Meteorology–Chemistry Interface Processor) converted meteorological model output into the CMAQ input format. The meteorological model has a vertical resolution of 40 terrain-following geometric height levels up to 22 km. IFS-CAMS cycle45r1 (Integrated Forecasting System—Copernicus Atmosphere Monitoring Service) [91] was applied as the Boundary Condition driver, with a vertical resolution of 60 sigma levels up to 65 km. CMAQ simulates 30 vertical levels, with the lowest layer ranging from 0 m to 42 m and the second layer ranging from 42 to 85 m.

Two simulations were performed with CMAQ: one run with the ship emission data as provided by STEAM and used in Fink et al. [38,61] and a second run with the ship emissions of the VOC groups adjusted by the adjustment factor derived from MAFOR, as described in Section 2.4, next. The simulated output was afterwards compared with observations at 28 monitoring stations distributed over the domain and as described in Fink et al. [61].

### 2.4. VOC Emission Factor Scaling

In the following, the adjustment procedure for one VOC EF, independently from volatility, for the ship emissions will be explained. This is done based on the reference plume on 03 September 2021 and by running different sensitivity cases with MAFOR. Toluene, xylene, trimethylbenzene, propane, ethane, and ethylene are VOCs used in MAFOR. The MAFOR model, furthermore, contains three volatility classes for primary delayed organic aerosols represented by PIOV, PSOV, and PELV [52]. Concentrations for PIOV, PSOV, and PELV at 530 m are 0.026 µg/m^3^, 0.036 µg/m^3^, and 0.25 µg/m^3^. These classes contain the condensing VOCs with low vapor pressure. Sulfate in MAFOR is condensed and the emissions of aromatics are oxidized and also contribute to the growth of particles. The formation of secondary aromatic aerosol in the particle phase is neglectable and is not displayed in the investigated time scale. The semi-volatile organic aromatics remained in the gas phase with a concentration at 530 m of 1.64 × 10^−4^ ng/m^3^. The input to initialize the MAFOR run is shown in Table A4.

The uncertainties of VOC emissions in ship emission inventories are large. They can range from 0.11 g/kg fuel to 6.93 g/kg fuel [53,56,57,58,59,85,86,87,88].

**Figure 3:**
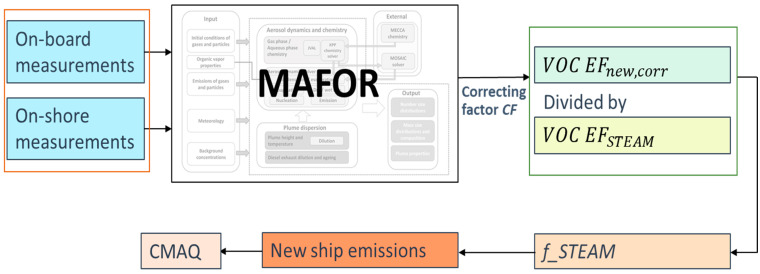
Approach for receiving ship emissions with adjusted VOC EFs.

The approach to receive a new VOC EF is presented in Figure 3. The NO_x_ emission factor *NO_x_ EF_new_* was the only EF that had been measured on board the ship by the Aeromon device. Therefore, all emission factors (EF_all_) were normalized to the initial NO_x_ EF (Equation (2)). The initial *VOC EF_init_* received from the literature was multiplied by the normalizing factor *NF_NO_x_* (Equation (3)):

(2) $$NF\text{\_}NO_{x}=\frac{{NO}_{x\;}{EF}_{new}}{{NO}_{x\;}{EF}_{init}}$$

(3) $$NF\text{\_}{NO}_{x}*VOC\text{\_}{EF}_{init}=VOC\text{\_}{EF}_{new}$$

The particle size distribution obtained from MAFOR simulations for the plume at the on-shore site for different VOC concentrations was evaluated by comparing against the measured particle size distribution and mean diameter, as described in Equation (1).

To investigate changes in the model performance in connection with the change in VOC emission factor, several sensitivity cases were conducted (Section 3.3, Table 2). This was done because no VOC EF was available for the present plume, and it cannot be taken for granted that the changes in the EF for VOC (compared to the engine testing conditions) are related to changes in the NO_x_ emission factor. The sensitivity cases allowed us to derive a new (real-world) emissions factor for VOCs, *VOC EF_new_*, by multiplication with a correcting factor *CF*:

(4) $$VOC\text{\_}{EF}_{new}*CF=VOC\text{\_}{EF}_{new,\;corr}$$

The sensitivity cases are named after the used correction factor (i.e., 0.5 means *VOC EF_new_* ∗ 0.5) within a range from 0.5 to 5.0. With a changing VOC EF, the size distribution of particles changes in the model. With a higher VOC EF, there will be larger particles, which are formed primarily by the growth of smaller particles through condensation. The particle number size distribution computed by MAFOR was compared to the measurement results. The modeled PNSD (particle number size distribution) was divided into seven size classes (described in Section 3.3). The best-fitting CF value was chosen for the size classes S2–S5 by calculating the sum of absolute differences between MAFOR simulations to measurements (Section 3.3, Table 2). These size classes (S2–S5) were chosen since they contain the most sensitive values. The optimal CF is the value where the sum of absolute relative differences is the lowest. The *VOC EF_new,corr_* with the lowest deviation from simulations to measurements serves as a base for comparison with the EF used in STEAM.

The emission factors for VOCs from MAFOR were obtained from measurements on the ship, operating at 50% engine load. Since the aim was to derive a new VOC emission factor for the STEAM emission dataset, the EF used in STEAM for VOCs for vessels at 50% engine load had to be related to the EFs for VOCs that were found in the optimization procedure using MAFOR (i.e., *VOC EF_new,corr_*). It is further assumed that the ratio of the *VOC EF_new,corr_* to the VOC EF_STEAM_ remains the same for all engine loads. The ratio between the two emission factors was calculated and the STEAM ship emissions for VOCs were adjusted by this factor, *f_STEAM*, for the CMAQ run:

(5) $$f\text{\_}STEAM=\frac{VOC\;{EF}_{new,\;corr}}{VOC\;{EF}_{STEAM}}$$

The STEAM data were used as ship emissions and for the comparison run with the regional-scale chemical transport model CMAQ, which is described in detail in Section 2.3.

## 3. Results and Discussion

### 3.1. Dilution of Gases and Particles between Ship and Remote Monitoring Station

To obtain more representative ship emissions for use in regional air quality models, the emission process from the stack to the background air needs to be considered in detail. Therefore, the emission process, e.g., in-plume chemistry and aerosol dynamics, should be addressed due to the extremely nonlinear reactions among gases and particle coagulation [47].

In addition to particle nucleation, condensation, and coagulation, dry deposition of particles, and gas-phase chemistry inside the plume, the model considered the mixing of air parcels with gases and particles in the background air.

**Figure 4:**
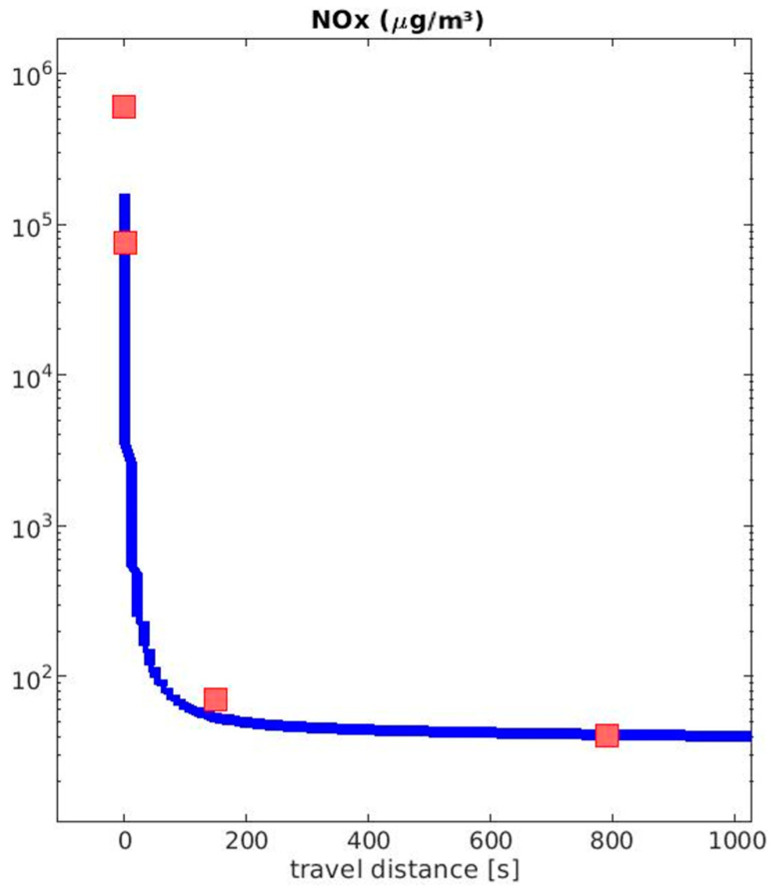
Dilution of NO_x_ on logarithmic scale; dark red dots are the measured NO_x_ values. Stack, measured by Aeromon BH-12 with an uncertainty of the measurement device of 19.7%, stack with 1:8 dilution, mobile lab, measured by airpointer 4D measurement system, background, measured by airpointer 4D measurement system with an uncertainty of 1 ppb (2 µg/m^3^).

NO_x_ dilution simulated by MAFOR suitably agrees with the observations at the sniffer location and the background concentrations (Figure 4). The dilution curve of NOx serves as a base for the dilution of other chemicals. Particles in the MAFOR model evolve after the model’s starting time of one second after the exhaust leaves the stack.

As described in Section 2.1, the peaks in the NO_x_ concentration and the particle number concentration at the shore-based sniffer station were measured at 17:41:00 UTC (Appendix A). The peak of NO_x_ was followed by a second peak at 17:42:00 UTC. A second peak was also observed in the particle number concentration, with the first peak appearing at 17:41:37 and the second one at 17:42:42 UTC. There is a delay in instrumentation and the instrument times were synchronized using the timing of the peaks. The aerosol measurements were obtained at a high temporal resolution, and the aerosol instrument response time was shorter than that of the NO_x_ sensor. Thus, the exact peak time could be slightly shifted and recorded. The explanation of the two peaks is the meandering behavior of the ship plume. In addition, the ship was moving, and the plume released one minute after the first plume may follow a different path.

### 3.2. Aerosol Size Distribution

**Figure 5:**
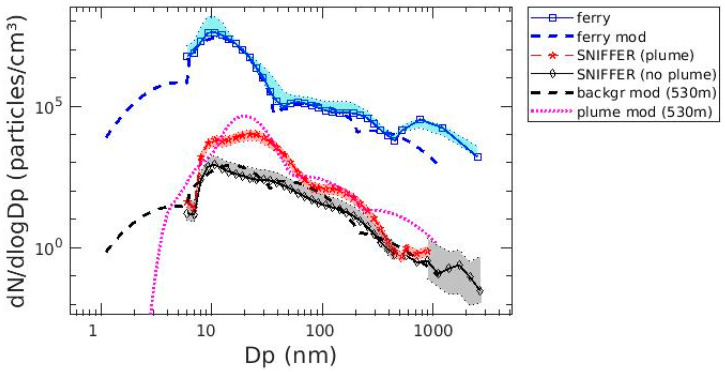
Comparison of the modeled particle size distributions with the observed particle size distributions. “ferry” = measured particle size distribution at the stack, with the shaded blue area indicating the 25th and 75th percentile. “ferry mod” = simulated particle size distribution at the stack. “SNIFFER (plume)” = particle size distributions recorded at the sniffer at the plume arrival with the shaded red area indicating the uncertainty range of ±40%. “SNIFFER (no plume)” = particle size distributions recorded at the sniffer without a plume event, with shaded grey area indicating the 25th and 75th percentile. “backgr mod (530 m)” = simulated particle size distribution at the sniffer without a plume event. “plume mod (530 m)” = particle size distributions simulated at the sniffer at the plume arrival.

MAFOR was used to calculate the changes in the exhaust particle size distribution over time with increasing downwind distance from the ship stack. MAFOR was adopted to simulate the particle size distribution starting with the input mass concentrations (Table 1). The measured particle background concentrations were observed at the same station as the plume measurements but at a different time. The station was located 530 m distant from the shipping lane. As shown in Figure 5, there is a number concentration peak in the Aitken mode for the measured background particles, indicating a certain influence of shipping-related emissions or other sources of UFPs.

In this study, one peak was found at 15 nm (Figure 5). Regarding the plume at the sniffer location (on-shore mobile lab), only the measurement with the highest concentration was considered; thus, no percentiles are displayed.

The EF for VOCs was adjusted as described in Section 2.2, and the amount of converted H_2_SO_4_ from SO_2_ was set according to the concentrations retrieved from Karl et al. (2020) [47]. With these settings, the simulated plume particle size distribution at a 530 m distance was optimized to narrow the discrepancy with the measured data at the sniffer location (Figure 5, red line). The optimized curve after using the correction factor is displayed in Figure 5, magenta line. This was done to obtain a correction factor by comparing several scenarios of the modeled output against the measured data from the sniffer station in regard to the number concentration and size distribution (Table 2). Only slight variations in the measurements on the ferry and at the mobile laboratory were found, as revealed by the 25th and 75th percentiles of the median of each bin for the measured data at the ship location and at the sniffer location without a plume (Figure 5).

It has been previously shown that the particle number size distribution of fresh diesel exhaust exhibits a bimodal character [47]. The measured size distribution at the sniffer station (Figure 5) exhibits a bimodal structure, indicating that the plume concentration has not yet reached the background concentration. Similar to this study, Pirjola et al. [43] found one dominating peak for the number size distribution in the Aitken mode, with one predominant size distribution in two modes and the dominating mode peaking from 20 nm to 30 nm. Nevertheless, the position of the maximum varied between 30 and 50 nm depending on the type of vessel; thus, comparing the results of this work with those of previous studies is hardly possible.

Karl et al. [47] applied the emission factor for the particle number concentration obtained by Moldanova et al. [45] directly to exhaust flow. However, no data were obtained from measurements on the ship at the same time as those on land, as was done in the present investigation.

### 3.3. Comparison between Simulated and Measured Data

In the common size class distribution as in Karl et al. [47,52], the integral number concentration and mean diameter of the modeled data were compared to those of the measured data (Table 2).

The best fit was selected based on the differences in size classes S2 to S5. Larger size classes were not used because of the high uncertainty caused by the fluctuations in the effective density of soot particles that could generate a systematic error of approximately 20% in PM estimation using the ELPI [92]. Regarding the small size classes (<15 nm), particle measurements suffer higher uncertainty due to the lower particle charging efficiency or higher diffusion loss [23].

The smallest total difference between the measured and modeled values was found when multiplying the initial VOC EF by a factor of 3.6 (Table 2). The best match for the number concentration and mean diameter could yield different CFs. Thus, the smallest total difference was based on the sum of the absolute difference of the relative deviation for the number concentration plus the sum of the difference of the relative deviation for the mean diameter in size classes S2 to S5 under the respective sensitivity case.

The evolution of the particle size distribution in the ship plume was found to be very sensitive to changes in the VOC EF. The relative differences between the simulated and measured size distribution data could either be positive (simulated values < measured values) or negative (measured values < simulated values).

If there was under- or overestimation of the VOCs, there could be a shift in the peak and a change in the distribution of the particles in the various size ranges. This occurred because all VOC EFs were adjusted, i.e., all volatility classes were adjusted. This also resulted in different deviations (positive or negative) in size classes S1 to S7.

### 3.4. Validation of New VOC EF_new,corr_

The new VOC EF _new,corr_ obtained for the reference plume was tested against 12 additional on-land measurements of plumes from the same vessel, with the assumption that the ship emissions and dilution conditions would remain the same (Table A5, Figures S1–S13). This leads to the possibility to add the confidence interval of the VOC EF, based on the range of the measured and simulated number concentration of each plume. This resulted in a VOC EF_new,corr_ of $0.323_{-0.1}^{+0.6}$ g/kg. The difference in number concentration shows that large size classes (S6 and S7) are overestimated by the model, whereas most middle (S3–S5) and small size classes (S1 and S2) are underestimated. The underestimation of nucleation mode particles indicates that the binary homogeneous nucleation process may not efficiently explain new particle formation in ship plumes. Non-volatile particles below 7 nm in size [93] forming in the hot exhaust, so-called core particles, may further grow by the condensation of volatile hydrocarbons and sulfur compounds during cooling [94]. Heterogeneous nucleation onto the solid core particles may play a role [95]. Arnold et al. (2012) [96] have observed condensable dicarboxylic acids in the exhaust of heavy-duty diesel vehicles equipped with modern exhaust after-treatment systems, which can contribute to the atmospheric nucleation processes. Pirjola et al. (2015) [97] investigated the potential of several nucleation mechanisms to predict particle formation in diesel exhaust and found that the best fit with particle size distribution measurements was predicted by a heteromolecular nucleation mechanism in which both sulfuric acid and semi-volatile organic acid molecules participate. However, nucleation is extremely dependent upon dilution conditions in the ship exhaust plume, and the measurement of small particles may be strongly influenced by diffusive losses and sampling artifacts, which makes it impossible to draw firm conclusions on the nucleation processes.

The pattern slightly differs for the mean diameter, where there are more cases of overestimations by the model. In general, the deviations between simulation and measurement for mean diameter are smaller than for number concentration.

It is generally assumed that wind speed has a large influence on dilution, so it was considered in detail for the 13 plumes.

At higher wind speeds (increase of 1 m/s and 2 m/s), the underestimation of the number concentration decreased, but only to a small extent (Figures S1–S13). This indicates that a change in wind speed has only a small impact on the aging in the current investigations. Celik et al. [98] found in a measurement study in the Mediterranean Sea that particle mass emission factors decrease (less condensation) as wind speed increases (stronger dilution), while particle number emission factors increase (less coagulation). They showed that the particle number in the plume was significantly influenced by wind speed. In the presented simulations of this study, changes in wind speed do not translate into different plume dispersion. Therefore, the apparent dependence of particle number concentration on wind speed in [98] is rather caused by different prevailing dilution regimes in the plume dispersion (slow dilution at low wind speed, fast dilution at high wind speed).

### 3.5. Application of Adjusted VOC Ship Emissions in a Regional-Scale CTM

In the following, the results of the ship emissions used in a regional-scale model are displayed to see whether there is a general improvement in the correlation coefficient as well as normalized mean bias (NMB) between simulated and measured PM_2.5_ data, when the aerosol modeling and the plume evolution are considered on a small scale.

By utilizing the adjusted VOC emission factor derived from the smallest-difference fit between the modeled and observed particle size distributions (Section 3.3), the ship emissions for the CTM run were modified.

The same adjustment factor (Equation (5)) was used to adjust the VOC emissions in all volatility classes. VOC emissions that have lower volatility and are partly in the condensed phase at standard conditions enter the CMAQ model as particulate organic carbon (POC) emissions. The volatilities of organic compounds in the CMAQ model are calculated internally based on the volatility basis set (VBS) approach [22,74], providing a framework for gas aerosol partitioning and chemical aging of both POAs and SOAs. The CMAQ model contains two basis sets for emitted primary organic aerosols, with each set containing five volatility bins: the first is for the directly emitted POA, while the second is for their multigenerational oxidation products [99,100]. Hence, the model redistributes the POC emission to species of the first volatility base set.

Although the CMAQ model includes many more (nonmethane) VOCs in the CB05 chemical mechanism, regarding the connection with the MAFOR model, only the POCs were modified, including semi- and low-volatile VOCs.

During the preprocessing of CMAQ emissions, the derived STEAM ship emissions were added to the land-based emissions, and the pollutants were assigned to the chemical species of CB05 in the CMAQ model. In this process, the STEAM VOC emissions were multiplied by the adjustment factor *f_STEAM* with a value of 18.13. This factor was received by dividing the VOC EF_new,corr_ of $0.323_{-0.1}^{+0.6}$ g/kg by the VOC EF_STEAM_ of 0.01779 g/kg fuel. The adjustment factor *f_STEAM* was applied to the four VOC groups in STEAM. The ship emissions of other substances derived from STEAM, as listed in Section 2.3, remained unchanged.

**Figure 6:**
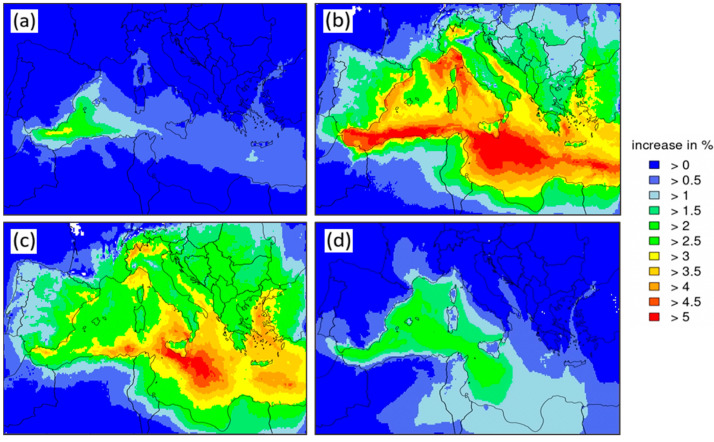
Changes in PM_2.5_ for the CTM run with adjusted ship emissions to initial ship emissions, displayed as mean values for (**a**) = March 2015; (**b**) = June 2015; (**c**) = September 2015; (**d**) = December 2015.

The CMAQ simulations with adjusted semi-volatile VOC ship emissions were compared to simulations without semi-volatile VOC emissions adjusted to study the effect on PM_2.5_ concentrations. The increase in semi-volatile VOC ship emissions by a factor of 18.13 caused an increase in the PM_2.5_ concentration along the major shipping routes by up to 5% (Figure 6). A detailed investigation on the individual aerosol species considered in the CMAQ aerosol module revealed that all showed changing values. The highest increase was found in anthropogenic semi-volatile primary organic compounds, particulate nitrate, and particulate chloride, with an over 40% increase in June. Thus, the scaling of POC emissions in CMAQ leads to particle growth and production of SOAs.

In this study, the highest increase was found in June (Figure 6c), with an increase in the total PM_2.5_ mass ranging from 3% to 5% over the sea. In the colder months of December and March with less solar radiation, the increase in the PM_2.5_ concentration varied between 0% and 2% (Figure 6a,d). There were no negative values. This indicates that the impact of VOCs on the background concentration of PM_2.5_ is relatively limited in the winter and early spring, which is similar to the results of the sensitivity study of Lee et al. [101] and can explained by decreased speed of chemical reactions at low temperatures. Also, emissions from ship traffic might be lower in winter. Absolute values in PM_2.5_ concentrations were mainly below 10 µg/m^3^ over water for both CMAQ simulations and indicate only slight differences in the run with adjusted and with original ship emissions (Figures S14 and S15).

**Figure A3:**
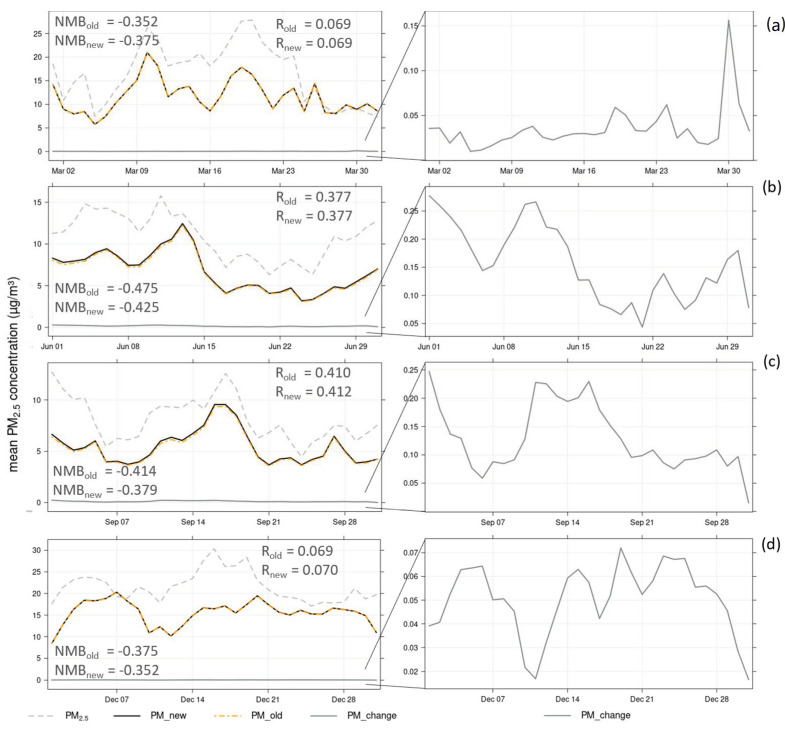
Time series at 28 monitoring stations over the whole domain; stations are described in Fink et al. [51]. (**a**) = March 2015, (**b**) = June 2015, (**c**) = September 2015, (**d**) = December 2015. R_old_ is the correlation for the PM_2.5_ concentration between measured and simulated concentrations for the run without the adjusted VOC emissions; R_new_ is for the run with the adjusted VOC emissions. NMB_old_ is the normalized mean bias for the PM_2.5_ concentration between measured and simulated concentrations for the run without the adjusted VOC emissions; NMB_new_ is for the run with the adjusted VOC emissions.

The change in PM_2.5_ daily mean concentrations remained well below 0.3 µg/m^3^, as revealed by the time series based on data at 28 monitoring stations (Figure A3). The correlation between measured and simulated data from the previous and the new run show that the change in VOC EFs at monitoring stations on land do not cause an increase in correlation coefficients with observed PM_2.5_. Also, the comparison of the NMB does not show any larger changes (not more than 5%); in all cases, the PM_2.5_ concentration was underestimated by the model. This is the case for all observed months (March, June, September, December; Figure A3). One might conclude from this that the distance to shipping lanes is either too large, so that the changes in PM_2.5_ are not observed at measurement stations, or that the effect of an adjusted VOC EF in ship emissions on total PM_2.5_ concentrations is rather small. Similar results were found in a study carried out in harbor city Marseille, where the ship impact on PM_2.5_ concentration was almost neglectable, whereas it was drastic for UFP numbers [48].

The overall change in PM_2.5_ concentration was small along the main shipping routes as well as at several harbor cities (i.e., Barcelona, Genoa, Nador, Oran, Algiers). Benchrif et al. [102] investigated PM sources in Moroccan harbor cities and found that the largest influence of PM in the Western Mediterranean Sea on air quality comes from maritime traffic emissions. In areas with increased PM_2.5_ concentration, they can have effects on radiative forcing and cloud formation.

## 4. Conclusions

The aerosol box model MAFOR was used to calculate the particle number and mass evolution in fresh ship plumes based on observed data received from a measurement campaign targeting particle emissions from a regular passenger ship. The model was validated by conducting several sensitivity runs with changes in the VOC emission factor to get the best approximation from modeled to observed particle size distribution data. The best-fitting approximation was used for correcting the emission factor of VOCs for ship engines. For the validation of the corrected VOC EF, 12 ship plumes monitored at the mobile lab on land were evaluated. This was done based on the assumption that each plume was homogenous and weather conditions were comparable during all 12 plumes that were measured on shore. With this, a confidence interval for the new VOC EF was calculated that indicates the range of the VOC EF and the variability that can occur due to the different conditions in the plumes and the environmental conditions. The newly determined VOC EF is confined by a confidence interval from −44% to 192%. The integration of the output obtained with the MAFOR model into the regional-scale model CMAQ was done via the ship emission dataset from STEAM by using the new VOC emission factor. Two runs with CMAQ were simulated, one with changes in ship emissions and one with the initial ship emissions. Results indicate that there exist effects of changes in VOC ship emissions on ambient PM_2.5_ concentration, but the overall changes in the concentration were rather small relative to the increase in VOC ship emissions. This low relative increase might be due to low levels of pre-existing particulate organic matter, which were present in the CMAQ simulations. This leads to generally lower particle formation. In the present study, only the lowest atmospheric layer was investigated. The concentration and amount of organic mass could be different in higher layers and deliver changed results.

Uncertainties in meteorology can also have an impact on the CMAQ simulations and the simulated concentrations, as already discussed in Fink et al. [38,61]. Even small changes in meteorological conditions (e.g., wind speed, humidity) might impact the particle formation processes.

Direct dilution of ship plume particles within the grid box volume of a regional-scale chemistry transport model leads to the underestimation of ambient particulate matter concentrations at coastal sites. This problem should be addressed in the future by integrating a sub-grid aerosol box model for the treatment of ship plumes in regional-scale chemistry transport models.

## Figures and Tables

**Table 1:** Mass distribution for background and ship aerosols; mass concentration is displayed in ng/m^3^; MSAp = methane sulfonate; PBA = primary biological material; v = volatile; nv = non-volatile; modes: NU = nucleation (Dp < 10 nm); AI = Aitken (10 nm < Dp < 50 nm); AS1 = accumulation 1 (50 nm < Dp < 500 nm); AS2 = accumulation 2 (>500 nm).

MODE	Dp (m)	SIG	H_2_SO_4_	OC	NH_4_	NO_3_	MSAp	SALT	PBA	EC	ASH	MTOT
			v	v	v	v	v	nv	nv	nv	nv	
**BACKGROUND AEROSOL**												
**NU**	9.90 × 10^−9^	1.70	0.001	0.002	0	0	0	0	0	0	0	0.003
**AI**	2.3 × 10^−8^	1.52	0.32	0.25	0.15	0.15	0	0.07	0	0.27	0.17	1.38
**AS1**	1.0 × 10^−7^	1.75	5	4	3	3	0	1	0	4	3	23
**AS2**	7.0 × 10^−7^	2.10	14	11	7	7	0	3	0	12	7	61
**SHIP AEROSOL**												
**NU**	9.6 × 10^−9^	1.65	0	0.026	0	0	0	0	0.011	0	0	0.05
**AI**	1.5 × 10^−8^	1.35	283	989.9	57	38	0	0	424.24	94.3	0	1886.14
**AS1**	1.3 × 10^−7^	1.75	1206	3657	241	161	0	0	1567.47	563	643	8038.47
**AS2**	7.5 × 10^−7^	2.10	10,726	32,535	0	0	0	0	13,943.78	3575	10,726	71,505.78

**Table 2:** Size ranges of the size classes. S1: 1 nm–10 nm; S2: 10 nm–20 nm; S3: 20 nm–50 nm; S4: 50 nm–100 nm; S5: 100 nm–300 nm; S6: 300 nm–600 nm; S7: >600 nm; differences are referring to the relative deviations in the modeled and measured data. CF = correcting factor., Because the CF is 1, 1.0 means no changes. The CF with the smallest deviation is shown in bold.

**Difference in Number Concentrations Compared to Measurements (in %)**								
**CF**	**S1**	**S2**	**S3**	**S4**	**S5**	**S6**	**S7**	**|S2| + |S3| + ** **|S4| + |S5|**
**0.50**	38.66	37.72	−206.51	3.63	−7.79	77.70	82.83	−172.94
**1.00**	−11.27	37.30	−117.90	3.91	−6.48	77.81	82.95	−83.17
**1.50**	−55.61	33.30	−69.65	4.16	−5.22	77.93	83.07	−37.41
**2.00**	−87.14	27.74	−40.20	4.41	−3.97	78.04	83.18	−12.02
**2.50**	−110.05	21.19	−20.72	4.68	−2.71	78.15	83.29	2.44
**3.00**	−128.98	13.76	−6.94	5.05	−1.46	78.26	83.40	10.41
**3.10**	−132.14	12.34	−4.90	5.13	−1.23	78.28	83.42	11.33
**3.20**	−136.16	10.46	−2.42	5.24	−0.94	78.31	83.45	12.33
**3.30**	−139.28	8.95	−0.57	5.33	−0.71	78.32	83.47	13.00
**3.40**	−142.35	7.39	1.19	5.42	−0.48	78.35	83.49	13.52
**3.50**	−146.31	5.37	3.31	5.56	−0.18	78.37	83.51	14.05
**3.60**	**−149.34**	**3.71**	**4.91**	**5.67**	**0.05**	**78.39**	**83.53**	**14.35**
**3.70**	−152.30	2.08	6.39	5.80	0.26	78.41	83.55	14.53
**3.80**	−155.24	0.42	7.80	5.93	0.49	78.43	83.57	14.63
**3.90**	−158.95	−1.71	9.48	6.10	0.75	78.46	83.60	14.63
**4.00**	−162.00	−3.53	10.82	6.26	0.98	78.48	83.62	14.52
**4.50**	−177.29	−13.13	16.62	7.16	2.02	78.58	83.72	12.67
**5.00**	−193.18	−23.97	21.42	8.37	3.10	78.69	83.83	8.93
**Difference in Mean Diameter Compared to Measurements (in %)**								
**CF**	**S1**	**S2**	**S3**	**S4**	**S5**	**S6**	**S7**	**|S2| + |S3| + ** **|S4| + |S5|**
**0.50**	8.55	6.66	10.53	−12.23	7.91	−3.32	6.23	162.50
**1.00**	10.35	3.10	12.30	−12.28	7.88	−3.34	6.21	137.99
**1.50**	11.58	0.29	12.63	−12.33	7.84	−3.35	6.19	117.65
**2.00**	11.49	−1.91	12.40	−12.37	7.80	−3.37	6.17	100.36
**2.50**	10.82	−3.59	11.88	−12.39	7.75	−3.38	6.15	85.21
**3.00**	10.04	−4.86	11.20	−12.36	7.70	−3.40	6.13	72.03
**3.10**	9.90	−5.05	11.07	−12.35	7.69	−3.40	6.13	60.29
**3.20**	9.73	−5.29	10.89	−12.33	7.68	−3.41	6.12	49.45
**3.30**	9.60	−5.46	10.75	−12.31	7.67	−3.41	6.12	39.75
**3.40**	9.47	−5.62	10.60	−12.30	7.66	−3.41	6.12	30.62
**3.50**	9.31	−5.81	10.40	−12.27	7.64	−3.42	6.11	21.99
**3.60**	**9.19**	**−5.96**	**10.25**	**−12.25**	**7.63**	**−3.42**	**6.11**	**13.93**
**3.70**	9.07	−6.09	10.09	−12.22	7.62	−3.42	6.10	13.93
**3.80**	8.96	−6.21	9.93	−12.19	7.60	−3.43	6.10	14.39
**3.90**	8.82	−6.35	9.73	−12.16	7.58	−3.43	6.10	14.39
**4.00**	8.70	−6.46	9.56	−12.12	7.57	−3.43	6.09	14.39
**4.50**	8.16	−6.88	8.67	−11.92	7.48	−3.45	6.07	20.30
**5.00**	7.63	−7.12	7.71	−11.64	7.38	−3.47	6.05	27.89

## Data Availability

The model output data and the measurement data used in this study will be made available upon request.

## Supplementary Materials

The following supporting information can be downloaded at https://www.mdpi.com/article/10.3390/toxics12060432/s1, Figure S1: Ship plume at 21.07.2021 17:31:10 UTC with changing wind speed. Figure S2: Ship plume at 02.08.2021 17:22:46 UTC with changing wind speed. Figure S3: Ship plume at 30.08.2021 17:34:00 UTC with changing wind speed. Figure S4: Ship plume at 01.09.2021 17:30:10 UTC with changing wind speed. Figure S5: Ship plume at 03.09.2021 17:41:40 UTC with changing wind speed. Figure S6: Ship plume at 08.07.2022 17:22:10 UTC with changing wind speed. Figure S7: Ship plume at 24.07.2022 16:28:20 UTC with changing wind speed. Figure S8: Ship plume at 26.07.2022 17:31:04 UTC with changing wind speed. Figure S9: Ship plume at 08.10.2022 16:29:49 UTC with changing wind speed. Figure S10: Ship plume at 19.07.2023 17:32:54 UTC with changing wind speed. Figure S11: Ship plume at 29.07.2023 16:40:50 UTC with changing wind speed. Figure S12: Ship plume at 26.08.2023 16:25:00 UTC with changing wind speed. Figure S13: Ship plume at 28.08.2023 17:28:10 UTC with changing wind speed. Figure S14: PM_2.5_ old absolute concentration, (a) mean value in March, (b) mean value in June, (c) mean value in September, (d) mean value in December. Figure S15: PM_2.5_ new absolute concentration, (a) mean value in March, (b) mean value in June, (c) mean value in September, (d) mean value in December.

## Appendix B

**Table A1:** Overview of the evaluated plumes. WD = wind direction (in degree), SD = shipping direction (in degree), WSP = wind speed (in m/s), SSP = ship speed (in m/s), Vector = calculated wind speed vector over the deck (in m/s), Travel = travel time of the plume from the ship to the sniffer (in s), RH = relative humidity (in %), Max. Meas. dN/dlogDp = maximum of measured dN/dlogDp, Max. Sim. dN/dlogDp = maximum of simulated dN/dlogDp in 530 m.

Date	Time	WD	SD	WDP	SSP	Vector	Travel	RH	Max. Meas. dN/dlogDp	Max. Sim. dN/dlogDp
**21.07.2021**	17:31:10	263 ± 16	14.8 ± 0.2	3.0 ± 0.6	6.3 ± 1.9	6.81	77.85	62.6 ± 1.6	4757.84	8196.38
**02.08.2021**	17:22:40	268. ± 12	17.0 ± 0.2	4.9 ± 1.1	6.7 ± 1.4	8.19	64.71	72.1 ± 1.4	3595.69	2540.21
**30.08.2021**	17:34:00	13. ± 19	37.9 ± 0.1	5.1 ± 0.8	6.6 ± 1.1	8.20	64.61	83.3 ± 1.6	10,054.93	2009.83
**01.09.2021**	17:30:10	290 ± 12	18.6 ± 0.2	3.2 ± 0.5	6.7 ± 1.8	7.37	71.90	72.2 ± 2.3	19,417.26	7751.90
**03.09.2021**	17:41:40	247 ± 10	18.3 ± 0.1	3.3 ± 0.6	5.4 ± 0.8	6.18	85.70	74.0 ± 1.3	10,283.38	12,648.08
**08.07.2022**	17:22:10	253 ± 13	16.1 ± 0.3	5.6 ± 0.9	6.6 ± 1.1	8.68	61.05	68.9 ± 1.2	5539.44	2194.99
**24.07.2022**	16:28:20	277 ± 22	17.7 ± 0.1	2.7 ± 0.9	6.2 ± 1.2	6.84	77.51	59.6 ± 2.8	7250.32	11,694.63
**26.07.2022**	17:30:30	276 ± 10	13.1 ± 0.1	5.8 ± 1.2	6.1 ± 2.2	8.33	63.60	57.3 ± 1.2	7350.02	2191.53
**08.10.2022**	16:29:30	258 ± 13	15.2 ± 0.2	4.8 ± 0.9	6.4 ± 2.1	8.03	66.04	71.7 ± 2.3	10,077.73	6174.34
**19.07.2023**	17:32:10	254 ± 15	17.4 ± 0.1	2.5 ± 0.5	5.3 ± 2.1	5.69	93.07	78.8 ± 1.8	2941.29	11,420.58
**29.07.2023**	16:40:50	267 ± 10	18.8 ± 0.2	5.1 ± 0.9	4.5 ± 2.4	6.89	76.91	70.1 ± 1.9	11,034.41	1453.65
**26.08.2023**	16:25:30	232 ± 9	14.6 ± 0.2	6.9 ± 0.8	6.0 ± 1.1	9.24	57.33	75.2 ± 1.4	6366.78	5407.44
**28.08.2023**	17:28:30	293 ± 13	17.5 ± 0.1	2.9 ± 0.7	5.9 ± 1.2	6.46	82.09	65.9 ± 1.6	9805.02	8832.01

## Appendix C

**Table A2:** Comparison of plume dispersion in MAFOR and Chosson et al. [67]. Unit for dil_rate_M_ is s^−1^ and for dil_rate_C_ is min^−1^.

MAFOR		Chosson et al. (2008) [67]	
Calculation	Parameters ^(1)^	Calculation	Parameters ^(2)^
${dil\text{\_}rate}_{M}={\left(\frac{a^{\prime }}{dil\text{\_}time}\right)}^{b}$	a′ = 1.659	${dil\text{\_}rate}_{C}={a(\frac{t*}{t})}^{b}$	a = 0.051 [min^−1^]
	b = 1.133		b = 1.08
	dil_time = input: time passed in plume [s]		t* = turn-over time scale [min]
			t = time passed in plume [min]

^(1)^ Based on adjustments from Moldanova, J. (personal communication). ^(2)^ Parameters are for a buoyancy flux of 250 m^4^/s^3^.

**Table A3:** Plume height and width approximation following the formulation of von Glasow et al. [68].

Plume Width		Plume Height	
$w_{pl}\left(t\right)=w_{0}({\frac{t}{t_{0}})}^{\alpha }$	*w_pl_* = plume width	$h_{pl}\left(t\right)=h_{0}({\frac{t}{t_{0}})}^{\beta }$	*h_pl_* = plume height
	*t* = plume time *t*_0_ *=* reference time after plume release *w*_0_ = reference dimension of the plume at time *t*_0_ α = plume expansion rates in the horizontal		*t* = plume time *t*_0_ *=* reference time after plume release *h*_0_ = reference dimension of the plume at time *t*_0_ *β* = plume expansion rates in the vertical

## Appendix D

**Table A4:** Procedure and intermediate steps to obtain the final input and starting concentrations for MAFOR from the emission factors of the individual substances. First-guess emission factors were received from the literature. The emission factor was multiplied by a changing factor of 0.67 to normalize to the emission factor of NO_x_, which was measured by Aeromon measurement device on board. Substance split was taken from the literature; for NO_x_ it was assumed that it consists of 90% NO.

Substance	First-Guess Emission Factor (g/kg_fuel)	Emission Factor (g/kg_fuel) Received with Changing Factor 0.67	Diluted Gases (g/m^3^) ^(1)^	Substance Split	Recalculation to cm^−^^3^	MAFOR Input after Dilution of 1/8 (in cm^−^^3^)	MAFOR Input VOCs without Dilution of 1/8 for VOCs (in cm^−^^3^) ^(6)^
**NO_x_**	40.91 ^(1)^	27.48	0.11	NO	2.041 × 10^15^	2.55 × 10^14^	
				NO_2_	2.268 × 10^14^	2.83 × 10^13^	
**SO_x_**	0.34 ^(2)^	0.23	0.00093	SO_2_	8.74 × 10^12^	1.09 × 10^12^	
				H_2_SO_4_	1 × 10^10 (4)^	1.25 × 10^9^	
				SO_3_	8.74 × 10^8 (5)^	1.09 × 10^8^	
**CO**	23.64 ^(1)^	15.88	0.065		1.40 × 10^15^	1.75 × 10^14^	
**HCHO**	0.0022 ^(1)^	0.0015	6.15 × 10^−6^		1.23 × 10^11^	1.54 × 10^10^	
**TOL**	0.0078 ^(3)^	0.0052	2.15 × 10^−5^		1.41 × 10^11^	1.76 × 10^10^	
**XYL**	0.00295 ^(3)^	0.002	8.15 × 10^−6^		4.63 × 10^10^	5.78 × 10^9^	
**TMB**	0.0028 ^(3)^	0.0019	7.68 × 10^−6^		3.85 × 10^10^	4.82 × 10^9^	
**IVOC**	0.0076 ^(4)^	0.018	7.56 × 10^−5^		2.53 × 10^11^		4.21 × 10^11^
**SVOC**	0.0105 ^(4)^	0.025	0.0001		2.51 × 10^11^		4.19 × 10^11^
**ELVOC**	0.105 ^(4)^	0.25	0.001		1.96 × 10^12^		3.27 × 10^12^

^(1)^ Based on Fridell et al., 2021 [72] (corresponding fuel mass flow: 0.00411 kg_fuel/m^3^). ^(2)^ Based on FSC limits. ^(3)^ Based on Wu et al., 2020 [53]. ^(4)^ Based on Karl et al., 2020 [47]. ^(5)^ Based on the assumption of 1% SO_3_. ^(6)^ Based on the concentration in cm^−3^ from Karl et al. 2020, which is already diluted; initial value is multiplied by 6 according to Wu et al., 2020 [53].

## Appendix E

**Table A5:** Size ranges of the size classes; S1: 1–10 nm; S2: 10–20 nm; S3: 20–50 nm; S4: 50–100 nm; S5: 100–300 nm; S6: 300–600 nm; S7: >600 nm; differences are referring to the deviations in the modeled and measured data. A CF (correcting factor) value of 3.6 was used in all simulations.

	**Difference in Number Concentrations Compared to Measurements (in %)**							
**Date**	**S1**	**S2**	**S3**	**S4**	**S5**	**S6**	**S7**	**|S2| + |S3| +** **|S4| + |S5|**
**21.07.2021**	69.95	62.16	66.19	−23.82	−1.11	47.24	95.05	153
**02.08.2021**	72.62	52.49	−77.02	−174.3	−71.18	70.1	−29.35	375
**30.08.2021**	−9.88	13.91	−192.51	−2.88	−3.7	75.3	71.62	213
**01.09.2021**	−0.33	18.67	0.25	−185.7	−60.78	56.9	99.2	265.4
**03.09.2021**	−149.34	3.71	4.91	5.67	0.05	78.39	83.53	14.35
**08.07.2022**	46.03	26.9	−234.71	−398.34	−156.89	42.85	90.65	816.83
**24.07.2022**	10.33	47.67	66.42	−25.36	−83.83	80.9	98.2	223
**26.07.2022**	−42.65	−51.75	−177.08	−92.6	−166.39	0.47	82.84	487.8
**08.10.2022**	54.22	44.05	−114.64	−144.76	−54.52	25.63	93.64	357.96
**19.07.2023**	90.85	70.46	86.88	9.08	18.33	23.3	97	184.8
**29.07.2023**	−74.36	−167.9	−704.88	−146.31	−40.31	−46.03	63.7	1059.4
**26.08.2023**	58.89	55.01	−27.51	−25.24	−57.76	48.32	91.98	165.52
**28.08.2023**	−4.94	−0.98	45.46	−19.47	−47.33	56.62	94.03	113.25
	**Difference in Mean Diameter Compared to Measurements (in %)**							
**Date**	**S1**	**S2**	**S3**	**S4**	**S5**	**S6**	**S7**	**|S2| + |S3| +** **|S4| + |S5|**
**21.07.2021**	−25.44	8.07	−14.77	14.15	−13.54	7.22	3.31	50.5
**02.08.2021**	−11.88	−2.3	−22.01	14.71	−4.4	34.46	1.02	43.43
**30.08.2021**	−42.84	−4.47	−16.13	14.25	−4.84	−5.87	−13.64	39.7
**01.09.2021**	−27.02	6.17	−15.8	14.35	−7.57	7.7	−2.55	43.89
**03.09.2021**	9.19	−5.96	10.25	−12.25	7.63	−3.42	6.11	13.93
**08.07.2022**	−14.22	−2.37	−22.88	18.31	−13.45	16.75	−3.02	57.01
**24.07.2022**	−10.4	8.86	−12	3.54	−1.3	14.43	−8.99	25.7
**26.07.2022**	−25.94	1.31	−14.26	12.43	−10.76	4.41	−10.26	38.8
**08.10.2022**	−16.65	−2.54	−20.73	17.50	−9.25	2.29	4.57	50.02
**19.07.2023**	−30.19	12.03	−13.41	6.74	−10.21	−2.91	2.68	42.38
**29.07.2023**	−16.6	−3.00	−14.73	14.15	−4.77	−3.87	3.22	36.63
**26.08.2023**	−25.28	−0.95	−16.17	2.84	−3.81	9.60	3.72	23.77
**28.08.2023**	−25.03	9.46	−9.56	9.42	−7.28	9.87	3.92	35.72
