# Supplementary material for: Using the Multicomponent Aerosol FORmation Model (MAFOR) to Determine Improved VOC Emission Factors in Ship Plumes
Source: Toxics. 2024 Jun 14;12(6):432. doi: 10.3390/toxics12060432 (PMC11209450; doi:10.3390/toxics12060432)
Supplement: Supplementary file 1 [file toxics-12-00432-s001.zip › toxics-3044708-supplementary.pdf]

toxics

Supplementary Material

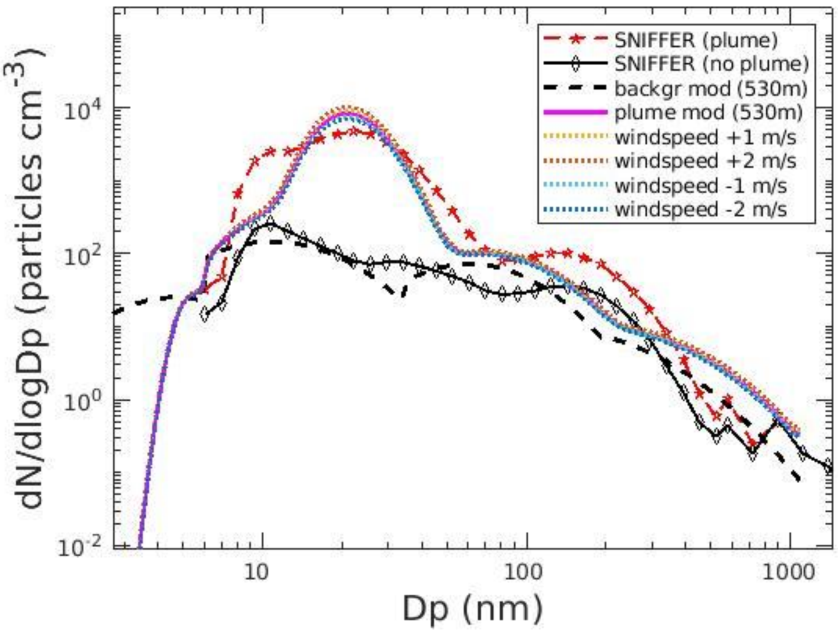

Figure S1: Ship plume at 21.07.2021 17:31:10 UTC with changing wind speed.

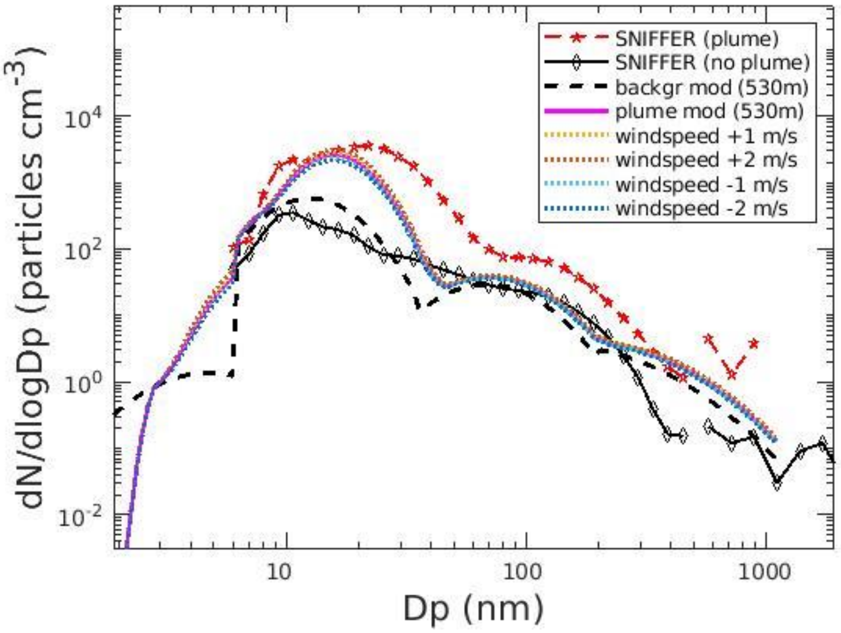

Figure S2: Ship plume at 02.08.2021 17:22:46 UTC with changing wind speed.

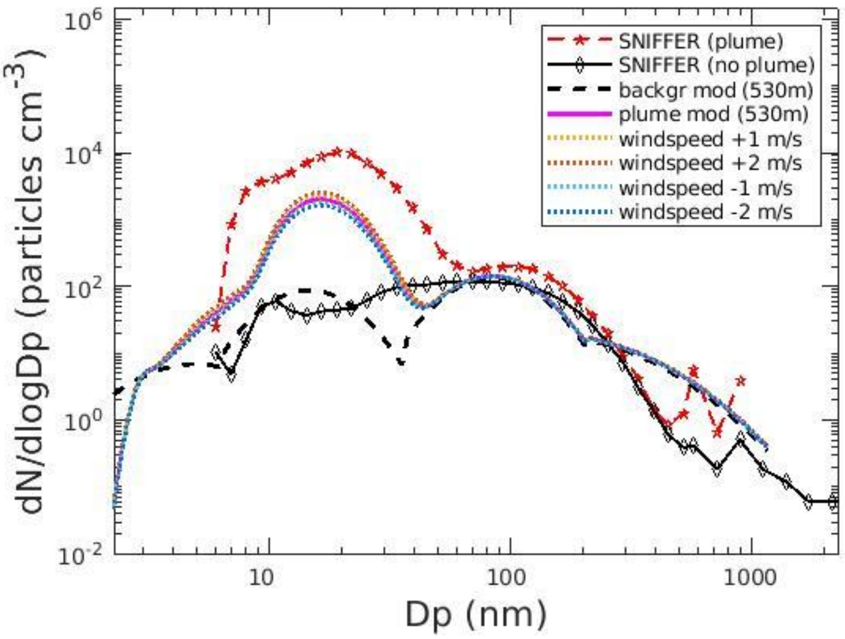

Figure S3: Ship plume at 30.08.2021 17:34:00 UTC with changing wind speed.

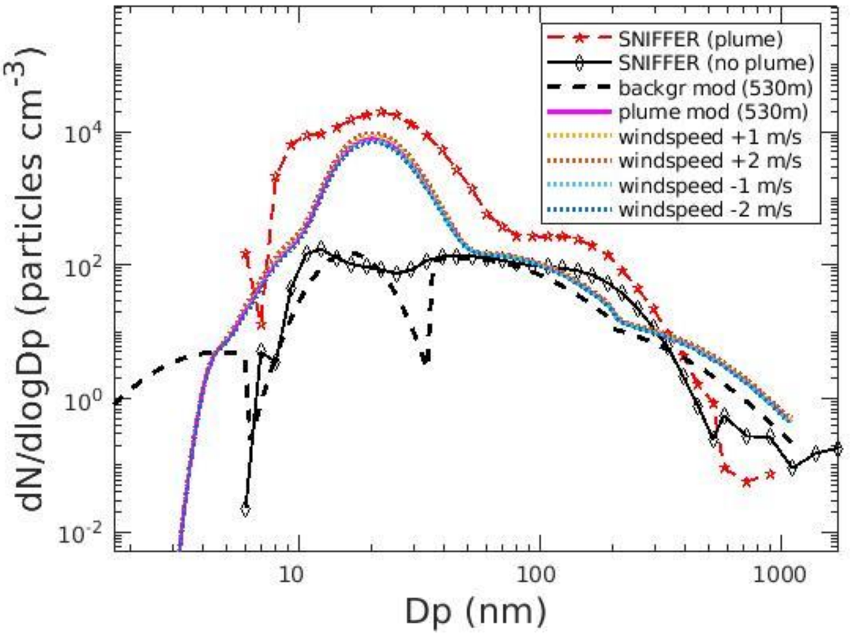

Figure S4: Ship plume at 01.09.2021 17:30:10 UTC with changing wind speed.

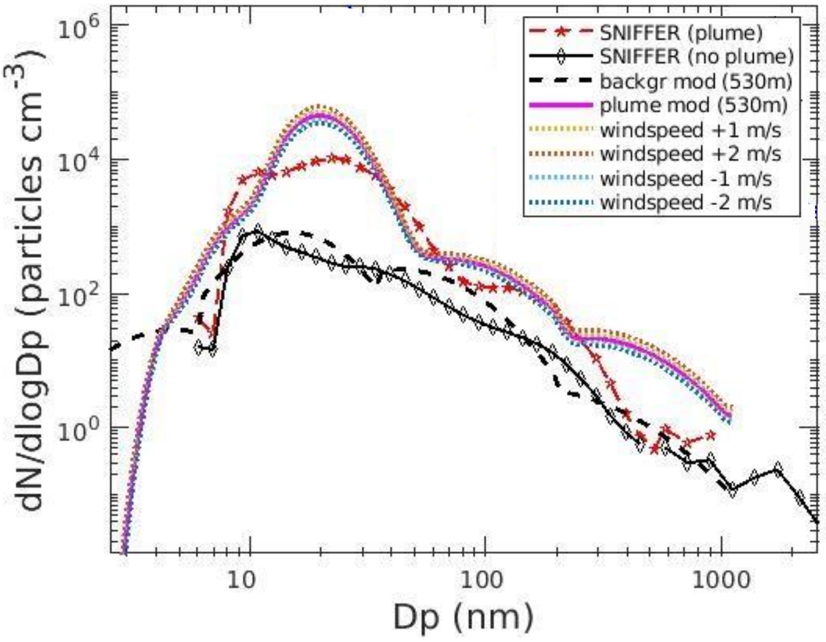

Figure S5: Ship plume at 03.09.2021 17:41:40 UTC with changing wind speed.

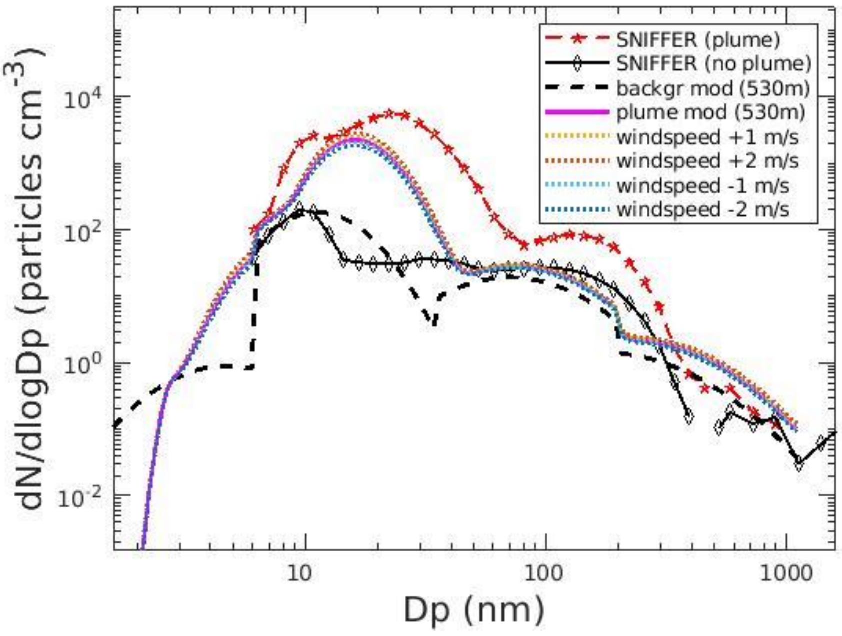

Figure S6: Ship plume at 08.07.2022 17:22:10 UTC with changing wind speed.

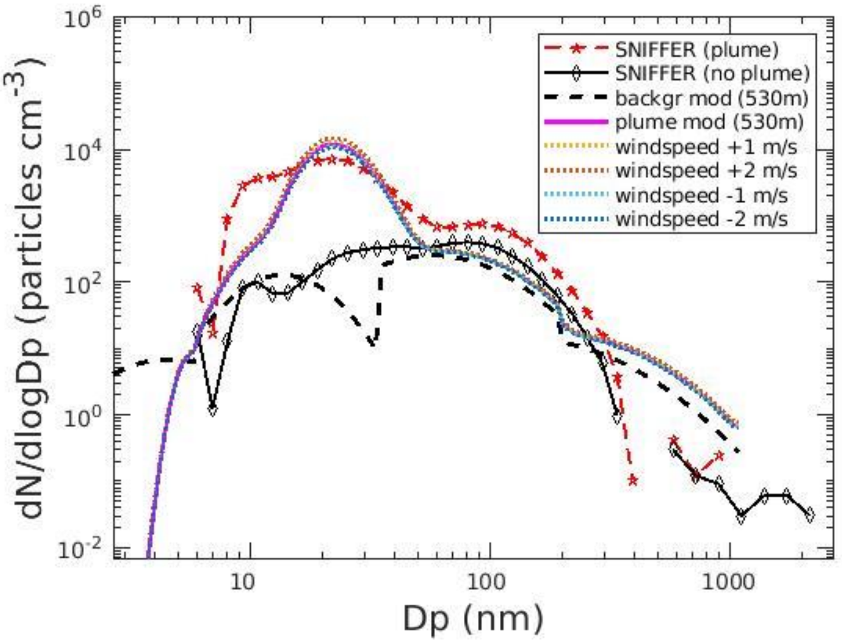

Figure S7: Ship plume at 24.07.2022 16:28:20 UTC with changing wind speed.

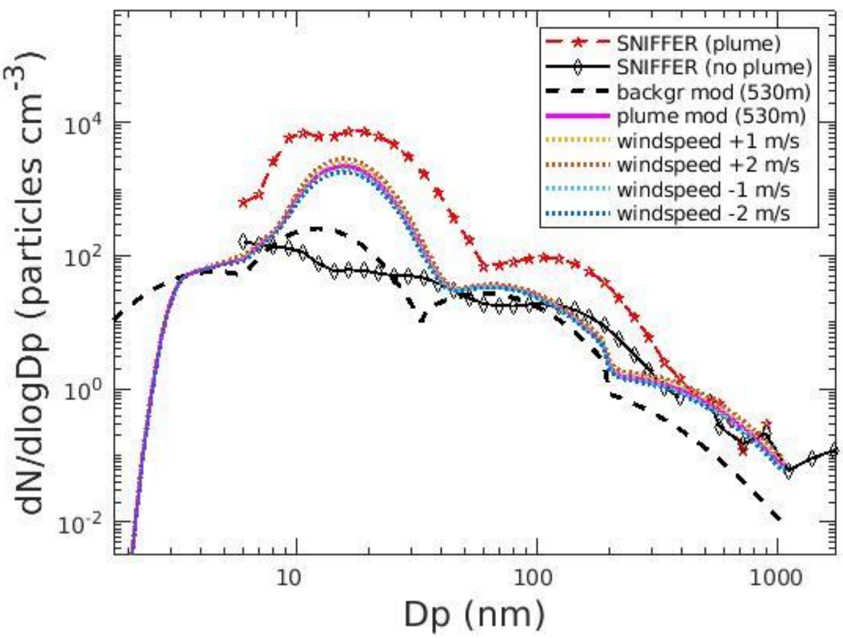

Figure S8: Ship plume at 26.07.2022 17:31:04 UTC with changing wind speed.

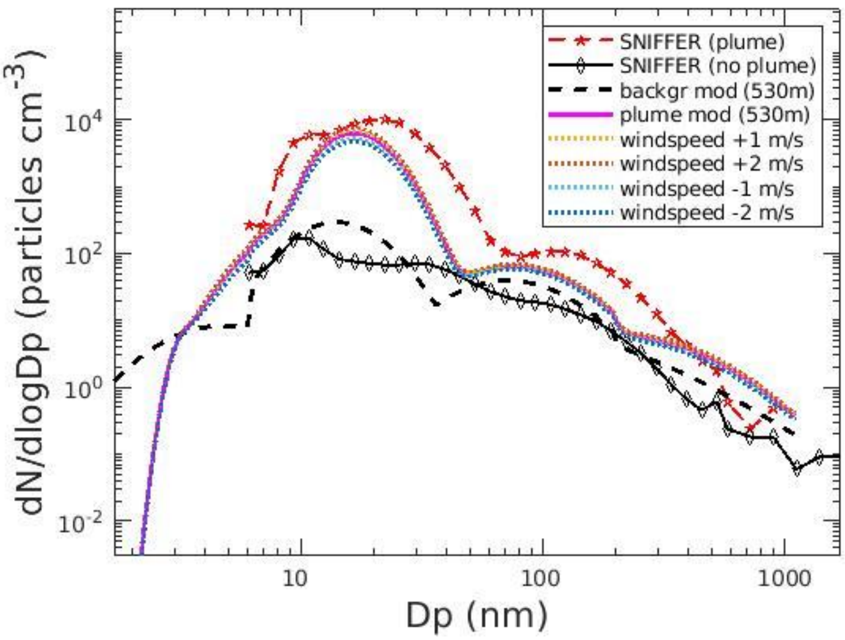

Figure S9: Ship plume at 08.10.2022 16:29:49 UTC with changing wind speed.

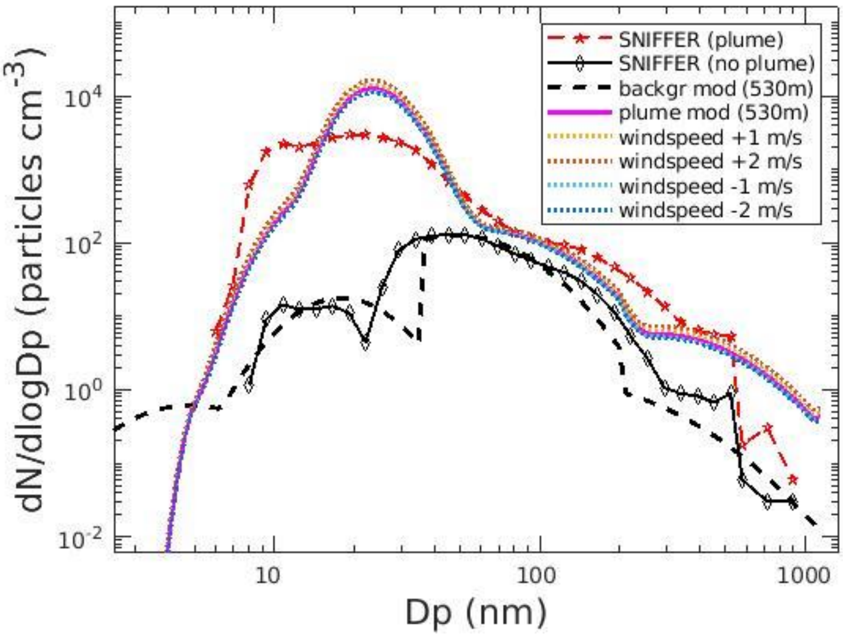

Figure S10: Ship plume at 19.07.2023 17:32:54 UTC with changing wind speed.

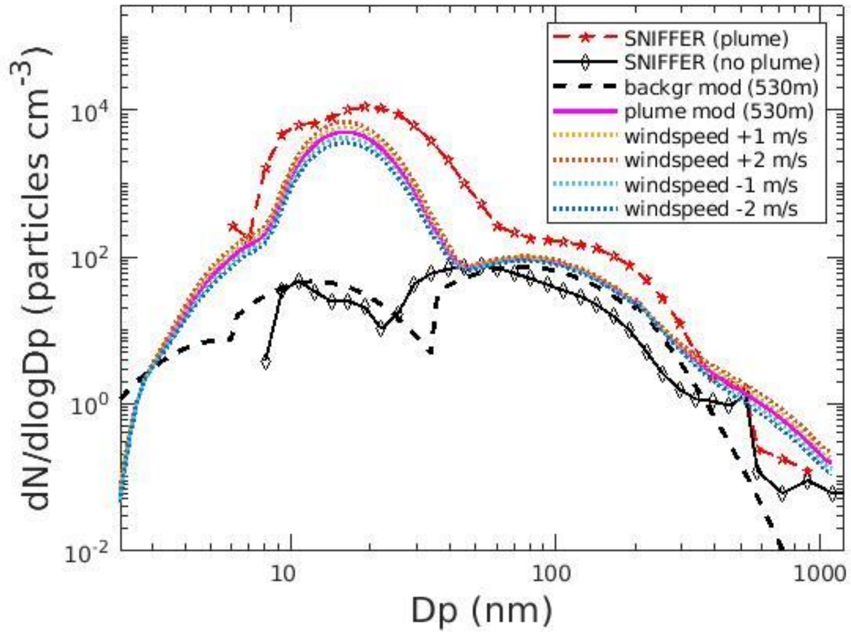

Figure S11: Ship plume at 29.07.2023 16:40:50 UTC with changing wind speed.

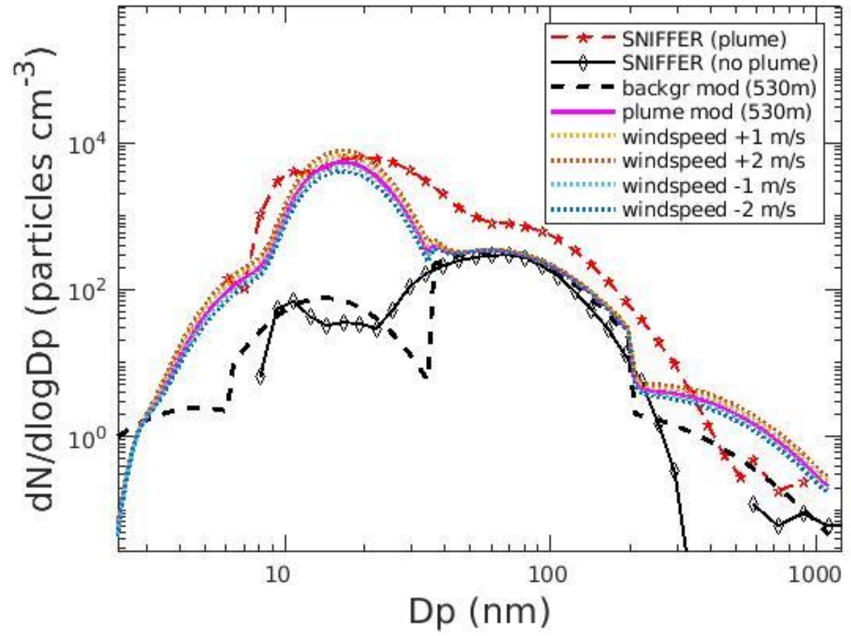

Figure S12: Ship plume at 26.08.2023 16:25:00 UTC with changing wind speed.

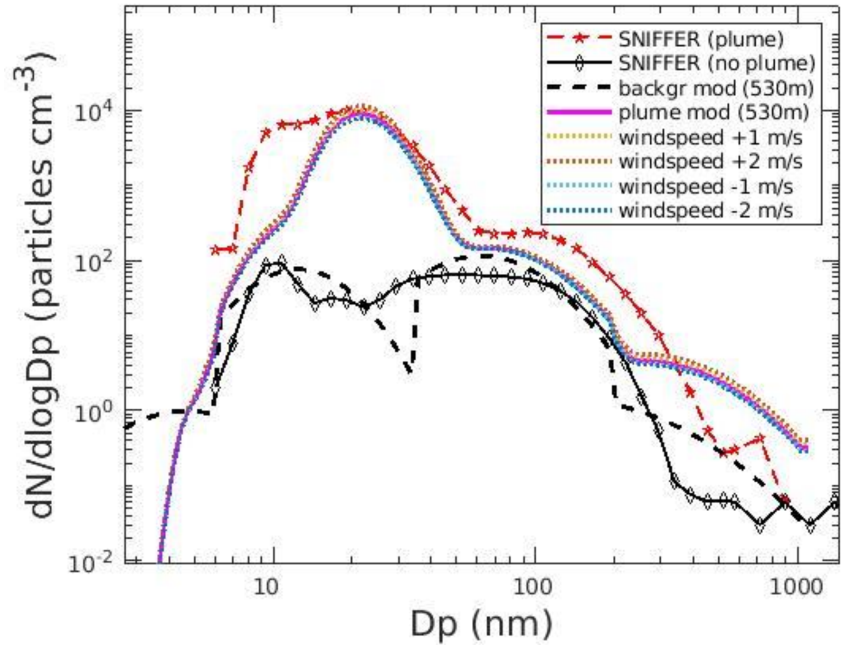

Figure S13: Ship plume at 28.08.2023 17:28:10 UTC with changing wind speed.

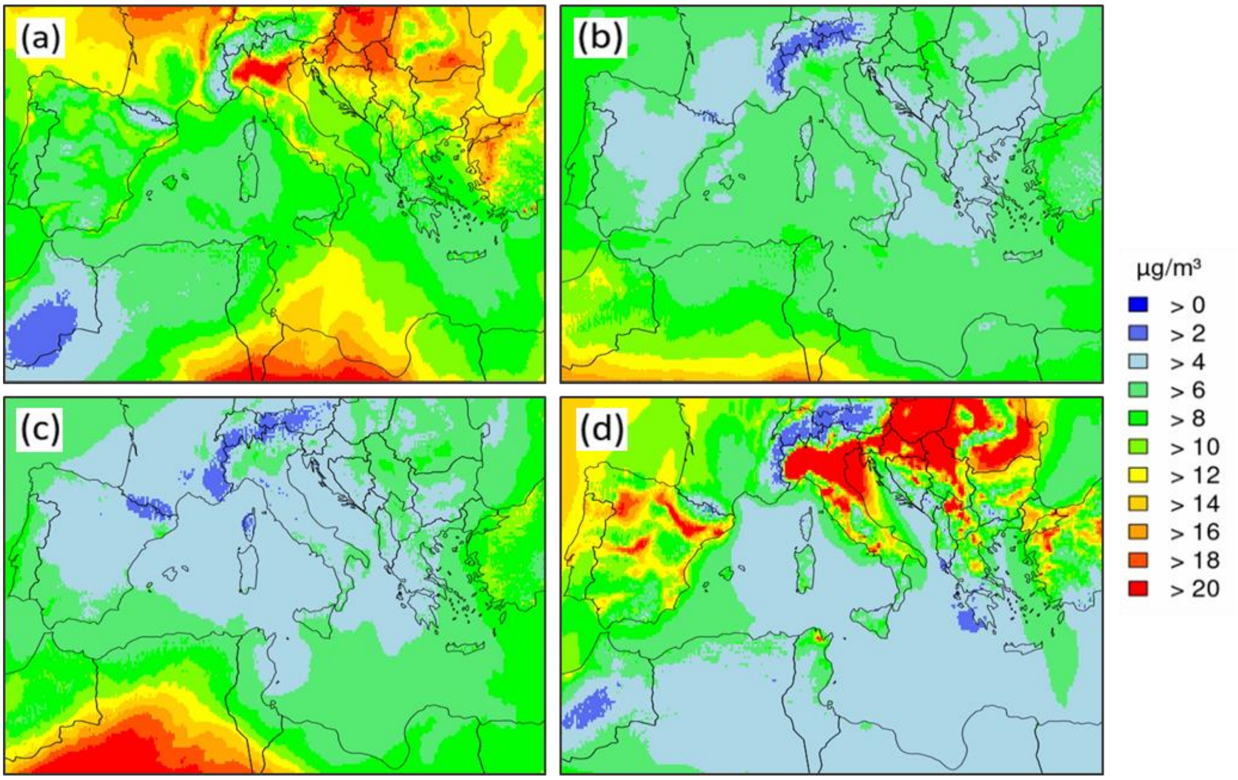

Figure S14: $PM_{2.5}$ old absolute concentration, (a) mean value in March, (b) mean value in June, (c) mean value in September, (d) mean value in December.

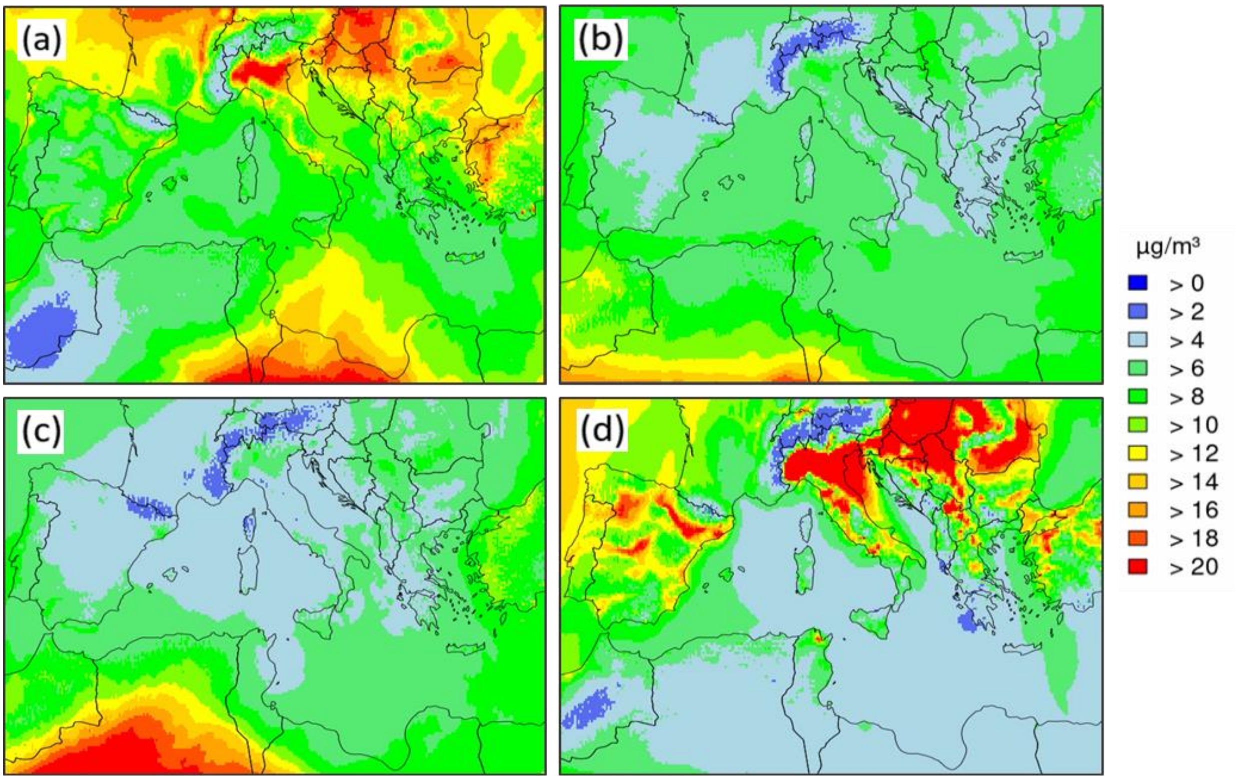

Figure S15: $PM_{2.5}$ new absolute concentration, (a) mean value in March, (b) mean value in June, (c) mean value in September, (d) mean value in December.
